# Introducing the Quality Target Administration (QTAP) Profile for Enteral Feeding Tube Medications

**DOI:** 10.3390/pharmaceutics18091089

**Published:** 2026-08-29

**Authors:** Smita Salunke, Chi Kin Anthony Chan, Sifan Hu

**Affiliations:** 1 Department of Pharmaceutics, School of Pharmacy, University College London, 29/39 Brunswick Square, London WC1N 1AX, UK; anthony.chan@student.kuleuven.be (C.K.A.C.); sifan.hu.17@ucl.ac.uk (S.H.); 2 Faculty of Pharmaceutical Sciences, KU Leuven, Herestraat 49, 3000 Leuven, Belgium

**Keywords:** Quality by Design, Quality Target Administration Profile, enteral feeding tube, critical administration attribute, pediatrics

## Abstract

**Background/Objectives:** The administration of medicines via enteral feeding tubes presents unique challenges, including dose loss, tube blockage, and user-dependent variability. Although Quality by Design (QbD) frameworks provide a structured approach to product quality, administration-related quality attributes are rarely assessed in a systematic manner during pharmaceutical development. A conceptual framework, termed the Quality Target Administration Profile (QTAP), was developed to extend QbD principles to the administration stage. **Methods:** The framework comprises three phases: administration context characterization, administration attribute identification, and risk-based determination of Critical Administration Attributes (CAAs). QTAP was applied to three representative EFT administration scenarios: a ready-to-use oral suspension, a crushed tablet, and an oral solution. **Results:** Application of the framework identified 18–32 administration attributes across the three representative scenarios and prioritized 5–6 CAAs per scenario according to clinical impact, variability risk, and human-factor considerations. Administration quality requirements varied across scenarios, reflecting differences in administration context, including dosage form, patient population, clinical setting, and user characteristics. QTAP provides a structured approach for identifying and prioritizing administration-related quality considerations within pharmaceutical development. **Conclusions:** As a conceptual framework, QTAP is intended to complement rather than replace existing QbD approaches. Future prospective validation, including human-factor studies and real-world evaluations, is needed to establish its practical utility and predictive value.

## 1. Introduction

Administration of drug products via enteral feeding tubes (EFTs) is a critical delivery route for patients unable to take medications orally, including those in intensive care units, individuals with chronic illnesses affecting swallowing function, and vulnerable pediatric populations. In the United Kingdom alone, approximately 17,000 children require nutritional and medication support through EFTs for survival [1]. The route is particularly prevalent in Neonatal Intensive Care Units (NICU), where immature coordination of sucking, swallowing, and breathing necessitates enteral access [2]. Despite this widespread clinical reliance, EFT medication administration remains a practice with quality and safety challenges that current pharmaceutical development frameworks inadequately address.

One of the fundamental challenges is a disconnect between product development and administration reality. Clinical practice frequently involves off-label manipulation of solid dosage forms such as crushing tablets, opening capsules, or improvising administration techniques due to the unavailability of suitable commercial products [3]. A study in a German hospital revealed that 46% of all medication manipulations were necessitated by administration via feeding tube [4]. These manipulations occur without systematic establishment of safety, efficacy, or administration performance, introducing substantial risks.

The consequences of inadequate attention to administration quality are well-documented in the clinical literature. Tube occlusion represents one of the most common and clinically significant failures, with an observational study reporting a 12.5% incidence of tube clogging with 10Fr and 12Fr nasogastric and nasointestinal tubes, attributed primarily to administration of solid-form medications [5]. Tube replacement necessitated by occlusion is traumatic for patients, particularly neonates and pediatric patients, disrupts therapy, and increases healthcare costs. In vitro studies have systematically demonstrated the mechanisms underlying these failures: tube clogging due to high viscosity formulations or large suspension particle sizes, incomplete dose delivery from product adherence to tube materials, and complications arising from gastrointestinal-intolerance-inducing excipients or interactions with enteral feeds and concurrent medications [6,7,8,9,10,11].

Beyond tube occlusion, dose variability presents a critical yet underappreciated quality failure. When tablets are crushed for EFT administration, dose losses of up to 38% can occur during transfer from crushing device to mixing container to administration syringe, yet this variability is rarely characterized during product development [12,13]. For narrow therapeutic index drugs commonly administered via EFT immunosuppressants in transplant patients, antiepileptics in neurologically impaired children, or cardiovascular agents in critically ill patients, such dose variability directly compromises therapeutic outcomes. Similarly, formulations not designed for EFT administration may exhibit unpredictable bioavailability changes when manipulated, altered pharmacokinetics from loss of modified-release characteristics, or enhanced toxicity from dose dumping when controlled-release tablets are crushed [14,15].

Healthcare professional challenges add to these product-related failures. Nurses, pharmacists, and caregivers face substantial confusion about appropriate administration techniques due to the absence of standardized guidance [16,17]. Questions raised during clinical practice include: which tablets are safe to crush? What flush volume ensures complete dose delivery for a given tube diameter? How should viscous suspensions be administered to minimize blockage risk? What constitutes adequate resuspension of a settled formulation? Current practice relies on empirical guidance documents such as the Handbook of Drug Administration via Enteral Feeding Tubes or institution-specific protocols, which categorize drugs based on anecdotal experience rather than systematic pharmaceutical characterization [18]. As a result, the same drug substance may be administered in different ways depending on local custom, clinician experience, or resource availability [10,16,19]. This knowledge gap places enormous responsibility on healthcare professionals to improvise quality-critical decisions without adequate manufacturer-provided data [20,21].

Recent regulatory guidance acknowledges these gaps. The European Medicines Agency (EMA) has emphasized the importance of providing robust data to demonstrate the feasibility of administering medicinal products via enteral feeding tubes, including evaluation of ease of administration, physical characteristics following manipulation, tube blockage risk, and dose recovery [22]. FDA guidance on oral products administered via EFTs already expects sponsors to consider in vitro tube testing, compatibility with enteral feeds and practical in-use conditions [23]. These reflect growing regulatory recognition that administration feasibility represents a quality domain requiring systematic attention. However, guidance on how to systematically assess and design for administration quality remains limited.

### 1.1. The Root Cause: Administration as an Afterthought

The recurring pattern across these failures reveals a fundamental gap in pharmaceutical development practice. Administration quality is addressed reactively, if at all, rather than proactively designed into products from the early development stages. Assessment of drug products for EFT administration typically occurs retrospectively, after formulation development is substantially complete. Products are evaluated for their suitability for EFT administration only after being optimized for their intended route (usually intact oral administration), creating an inherent mismatch between product characteristics and administration requirements [24].

This reactive approach defies a core principle articulated by the FDA: “quality should be built into the product, and testing alone cannot be relied on to ensure product quality” [25]. Drug formulations not originally designed for EFT administration may pass all standard quality tests in their intended form and meet specifications for content uniformity, dissolution, stability, and microbial limits, yet fail when manipulated for enteral delivery. For instance, a tablet with excellent dissolution characteristics for oral swallowing may produce particles too large for 8Fr neonatal feeding tubes when crushed [18]. A suspension with appropriate viscosity for direct oral administration may be too viscous to flow through small-bore tubes or may require flush volumes exceeding fluid restrictions in critically ill infants. These real-world performance failures occur despite products meeting all traditional quality specifications, highlighting that administration quality exists as a distinct area inadequately captured by conventional pharmaceutical quality frameworks. Additionally, administration through EFTs is influenced by many interacting variables, including tube characteristics, placement, patient populations, and flushing practices [24]. These factors vary widely across clinical and home settings and directly affect dose delivery and clogging risk. Together, they create a complex, multidimensional administration landscape that cannot be adequately addressed by product specifications alone.

### 1.2. Quality by Design: Foundation for a Solution

Quality by Design (QbD) represents the pharmaceutical industry’s paradigm shift from reactive quality testing to proactive quality design [26,27,28,29,30,31]. Central to QbD is the Quality Target Product Profile (QTPP), which outlines the quality characteristics essential to achieving the intended clinical outcomes [32]. These characteristics are then translated into Critical Quality Attributes (CQAs), which must be monitored and controlled throughout development [33].

Complementing QTPP is the Target Product Profile (TPP), a strategic template that articulates desired product characteristics including indications, target population, dosage form, dosing regimen, route of administration, and safety/efficacy targets. It includes both minimal and preferred product attributes and gives innovators a defined framework to evaluate their drug candidate as they move through the development process. Together, TPP and QTPP have evolved into dynamic documents bridging preclinical, clinical, regulatory, and commercial considerations across the drug development lifecycle.

However, while QTPP systematically addresses formulation composition and manufacturing quality, its application to administration quality remains underdeveloped. ICH Q8 guidance specifies that QTPP should include route of administration and dosage form as quality attributes [31]. However, it provides limited guidance on systematically dissecting these attributes into administration-specific quality elements. In practice, QTPP documents frequently list administration superficially without further elaboration of what quality characteristics ensure successful administration through this route. This gap is particularly acute for EFT products, where administration involves complex user–product–device interactions as discussed above.

### 1.3. The Need for a Quality Target Administration Profile (QTAP)

Current approaches acknowledge administration within QTPP but do not provide a comprehensive, systematic framework for translating administration requirements into measurable quality attributes, risk assessments, and control strategies. The literature and regulatory guidance highlight administration as an attribute but offer little structured methodology for dissecting this single attribute into its constituent quality elements [22,24]. There exists an opportunity indeed, a necessity to magnify the administration dimension through a dedicated QTAP that extends QbD principles from product formulation to administration performance.

A QTAP would systematically address questions currently left to clinical improvisation: What constitutes appropriate dose recovery for a given administration route and patient population? What tube compatibility characteristics should be specified? How should formulation parameters be linked to administration performance? What healthcare professional technique dependencies exist and how should they be managed? What patient-centric factors (caregiver burden, administration complexity, or error potential) represent quality attributes requiring design control? These questions remain largely unanswered in current pharmaceutical development practice, yet their answers directly determine therapeutic success or failure at the critical moment of administration.

The need for QTAP is particularly important for products likely to be administered via EFTs, especially pediatrics. Pediatric patients are vulnerable with narrow therapeutic windows, doses are weight-based with minimal margin for error, caregivers may lack medical training, administration occurs in unsupervised home environments, and tube blockage or dose delivery failures carry severe consequences.

This paper introduces the QTAP as a systematic framework for extending QbD principles to the administration domain, using enteral feeding tube medications as an exemplar of complex administration requiring rigorous quality design. The objectives are threefold:

1. To define the QTAP concept and establish its relationship to existing QbD frameworks.
2. To demonstrate QTAP application to three representative EFT administration scenarios to illustrate how the framework generates context-specific administration quality attributes and identifies Critical Administration Attributes (CAAs).
3. To explore the integration of QTAP into pharmaceutical development to support formulation design, testing strategies, and administration instructions.

## 2. Conceptualizing the Quality Target Administration Profile

From a QbD perspective, the QTAP is a systematic analysis of the administration element within the QTPP. It is defined as a prospective summary of the quality characteristics related to the administration of a drug product that collectively ensure successful dose delivery in real-world clinical settings. It ensures the intended dose reaches the patient safely, accurately, and in a manner that supports adherence and therapeutic success. QTAP provides a structured framework for translating broad statements, such as suitable for administration via enteral feeding tube, into specific, controllable characteristics such as particle size < 20 μm to prevent blockage in 8Fr tubes or flush volume ≤ 5 mL for dose recovery > 95% in pediatric patients. It specifically addresses the dynamic interplay between formulation characteristics, delivery method, user capabilities, device interfaces, and environmental conditions. For EFT medications specifically, QTAP includes but is not limited to attributes illustrated in Table 1.

The QTAP focuses exclusively on the CQAs that can be defined, measured, and controlled within the administration domain and that directly influence whether the intended dose is successfully delivered as designed. To maintain clarity, it is important to specify what falls outside the QTAP scope. The chemical and physical attributes of the formulation itself, such as drug substance purity, potency, stability, and dissolution characteristics, remain within QTPP. Manufacturing process parameters and in-process controls continue to be addressed through process design space and Critical Process Parameters (CPPs). Clinical outcomes beyond those directly attributable to administration quality, including disease progression, off-target pharmacological effects, or individual patient response variability, are not QTAP elements. Post-administration physiological processes such as absorption, distribution, and metabolism are not QTAP attributes per se, though QTAP does consider administration factors that influence these processes. For example, the rate at which a viscous suspension is administered through a feeding tube affects gastric emptying and may influence absorption kinetics [34]. This makes administration rate an appropriate QTAP consideration even though absorption itself remains a pharmacological rather than administration quality attribute.

### 2.1. QTAP in the Context of TPP and QTPP: The Quality Continuum

In established QbD practice, the TPP defines the intended clinical rationale and therapeutic outcomes, indication, target population, dosing regimen, route of administration, and key safety/efficacy claims. It articulates what therapeutic objectives the product must achieve and for whom. For an EFT medication, the TPP might specify:

- Indication: Treatment of infection in hospitalized pediatric patients.
- Target population: Neonates through children aged 12 years.
- Administration route: Oral or enteral feeding tube (for patients unable to take oral medications).
- Dosing regimen: Weight-based dosing, every 8 h for 7–10 days.
- Key clinical attributes: Rapid onset of action, broad spectrum coverage, and pediatric-appropriate safety profile.

The QTPP translates TPP clinical objectives into measurable product quality attributes [35]. The QTPP answers: What physical, chemical, and pharmaceutical characteristics must the product possess to achieve TPP objectives? This includes specifications for dosage form, strength, release profile, stability, and key quality attributes such as identity, purity, potency, and pharmaceutical elegance. The QTPP focuses on what the manufacturer controls and delivers the product as it exists at release and throughout its shelf life [35]. For the same EFT medication, QTPP would specify

- Dosage form: Oral suspension (to accommodate inability to swallow in target population).
- Strength: 100 mg/5 mL (enables weight-based dosing with reasonable volumes).
- Route of administration: Oral or enteral feeding tube.
- Drug substance characteristics: Identity, purity, and potency.
- Drug product characteristics: Appearance (uniform pink suspension), pH (5.0–7.0), viscosity, particle size, osmolality, and microbial limits.
- Stability: ≤5% degradation over 24 months at 25 °C/60% RH; in-use stability 30 days refrigerated after opening.
- Container-closure system: Amber glass bottle with child-resistant cap; oral syringe provided.

**Figure 1:**
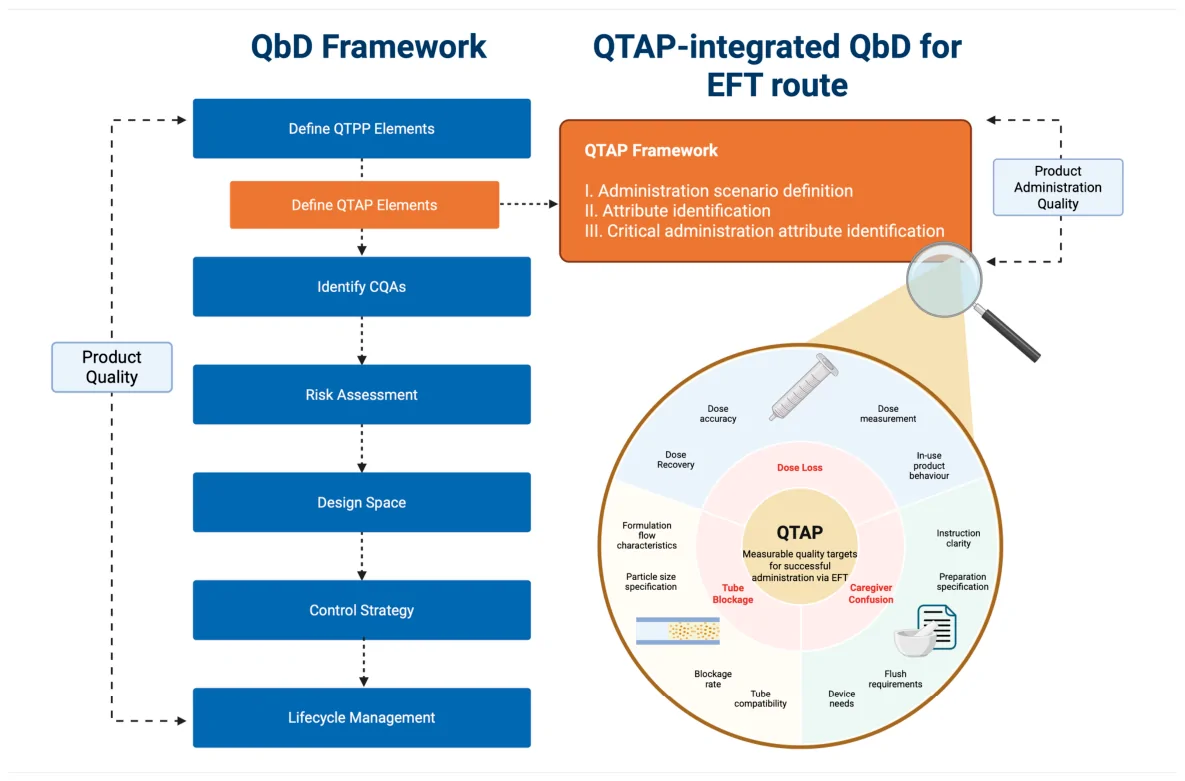
Illustration of the QTPP–QTAP magnification concept. Created in BioRender. https://BioRender.com/o4bkbv7.

Route of administration, dosing frequency, and basic usability already appear as attributes within TPPs and QTPPs, but they are treated as high-level descriptors rather than as full, systematic administration specifications. A typical QTPP entry states: “Route of administration: Oral or via enteral feeding tube.” While this acknowledges the EFT route, it provides no measurable quality targets for what makes administration via feeding tube successful. QTAP systematically magnifies this single QTPP attribute into its constituent quality elements (Figure 1). Each QTAP element addresses a documented failure mode from clinical practice: tube blockage, dose losses, and caregiver confusion.

QTAP is not separate from QTPP but rather a natural extension applying QbD thinking to fill the critical gap between product quality at release and dose delivery quality in practice (Table 2). Just as QTPP systematically translates clinical objectives into product specifications, QTAP systematically translates administration requirements into measurable, controllable attributes.

QTAP answers: What characteristics of the administration process ensure that the quality product defined by QTPP delivers the intended dose through the specified route to achieve TPP objectives? For this same medication:

- Tube flow characteristics: Flows through 8–24Fr feeding tubes within 2 min; no excessive resistance requiring > 10 N plunger force.
- Blockage prevention: Particle size distribution (d90 < 20 μm) prevents tube occlusion; suspension rheology enables flow without settling during administration.
- Dose measurement: Graduated oral syringe enables accurate measurement of weight-based doses (±5%); clear graduation marks visible to caregivers.
- Dose recovery: ≥95% of measured dose delivered through tube with 5 mL flush; <5% product adherence to tube materials or syringe.
- Suspension uniformity: Complete resuspension within 30 s of vigorous shaking; remains uniform for 2 min administration window.
- Administration time: Complete dose delivery (measure, administer, and flush) achievable in <5 min with trained technique.
- Instructions clarity: Shaking instructions, flush protocol, and troubleshooting guidance understandable to nurses and caregivers with varied health literacy.

### 2.2. QTAP for Enteral Feeding Tube Products

While the QTAP concept has potential application across diverse administration routes and patient populations, EFT medications present a particularly compelling case for systematic administration quality assessment due to the convergence of multiple complexity factors.

- **Administration device interface complexity**

Unlike oral administration where the drug product interacts solely with the patient, EFT administration introduces a device (the feeding tube) as an intermediary. This device is not controlled by the pharmaceutical manufacturer, and varies across institutions and patients (6Fr to 24Fr diameters, PU/silicone/PVC materials, 30–120 cm lengths), yet profoundly affects administration success [23,24,36]. Drug tube interactions, viscosity effects on flow, particle size effects on blockage risk and chemical interactions with tube materials create administration quality dependencies that do not exist for intact oral administration.

b. **Multi-step administration process with cumulative failure risk**

EFT administration involves sequential steps, each introducing potential quality failures: (1) accessing product from container, (2) shaking/mixing to achieve uniformity (for suspensions), (3) accurately measuring weight-based dose, (4) drawing into appropriate syringe, (5) connecting to tube port, (6) administering at appropriate rate, and (7) flushing to recover complete dose [37]. For crushed tablets, additional steps include: (8) crushing to adequate particle size, (9) transferring powder with minimal loss, and (10) mixing into suspendable slurry. Each step compounds variability. Incomplete shaking yields non-uniform dose; inaccurate measurement directly affects dose; insufficient flushing leaves drug in tube dead space; and inadequate crushing creates particles, causing blockage [24,36].

c. **Vulnerable patient populations**

EFT-dependent patients include clinically vulnerable premature neonates in the NICU, children with severe developmental disabilities, elderly patients with dysphagia, and critically ill adults [36,38]. These populations have limited physiologic reserve to tolerate dosing errors and high risk of serious consequences from administration failures. Weight-based dosing in pediatrics amplifies the impact of dose variability; a 20% dose loss that might be clinically insignificant in a 70 kg adult becomes unacceptable in a 3 kg neonate receiving narrow therapeutic index drugs. A delphi consensus study in pediatric critical care found that for most mediations, dosing more than 10% above or below the target range was considered a dosing error [39].

d. **Healthcare professional and caregiver technique dependency**

EFT administration quality depends heavily on user technique. The level of user training and experience varies widely across care contexts [14]. Technique variables such as adequacy of shaking, accuracy of measurement, completeness of flushing, and timing relative to enteral feeding directly affect dose delivery but are difficult to standardize [36]. This human-factor variability makes administration quality challenging to control through product design alone. The systematic QTAP assessment can identify attributes that minimize technique dependency (e.g., suspensions that remain uniform for extended periods reduce consequences of inadequate shaking).

e. **Regulatory and evidence gaps**

Unlike well-established routes such as oral tablets or injections where decades of experience inform formulation design, EFT administration lacks comprehensive quality frameworks. Both EMA recommendations and the FDA draft guidance acknowledge this gap by calling for data on tube blockage risk and dose recovery [22,23]. However, a harmonized framework for systematically identifying, prioritizing, and controlling administration-related quality attributes remains lacking. QTAP offers a structured approach to address these regulatory expectations.

f. **High failure rates in current practice**

As documented in Section 1, current EFT administration exhibits unacceptably high failure rates, frequent tube blockage incidence, significant dose losses during crushed tablet administration, widespread healthcare professional confusion about appropriate techniques, and frequent adverse events from drug–tube–formula interactions. These failures indicate that current ad hoc approaches to administration quality are inadequate. The magnitude and frequency of failures justify systematic intervention through frameworks like QTAP.

These factors collectively make EFT medications an ideal exemplar for QTAP introduction. If QTAP can systematically address the complexity of EFT administration, its principles can be adapted to simpler administration scenarios with greater ease. Conversely, addressing simpler routes first might produce frameworks that fail when confronted with EFT complexity.

## 3. QTAP Framework

### 3.1. Framework Overview

**Figure 2:**
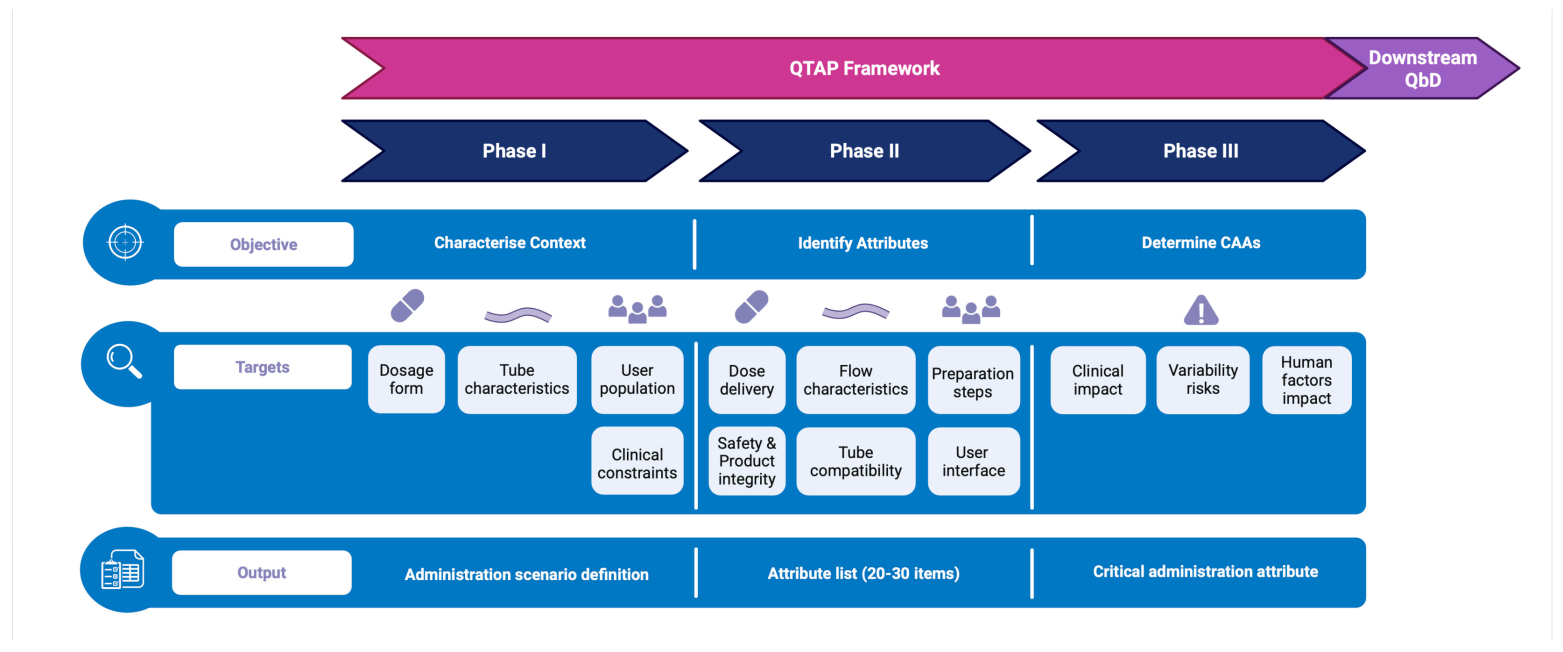
Overview of the three–phase QTAP framework: context characterization, attribute identification, and CAA determination. Created in BioRender. https://BioRender.com/o4bkbv7.

The QTAP framework was developed as a structured methodology for identifying and prioritizing administration-related quality attributes. The approach comprises three sequential phases: (i) administration context characterization, (ii) administration attribute identification, and (iii) risk-based determination of CAAs, adapted from ICH Q8/Q9 principles for quality attribute identification and risk assessment (Figure 2). The proposed attribute categories and criticality criteria were developed through a structured process integrating a review of the EFT-specific literature, including reports of in-use performance testing and real-world clinical practice, relevant regulatory guidance on formulation development for EFT administration, and the authors’ expert judgement based on their pharmaceutical development experience.

#### 3.1.1. Phase 1: Administration Context Characterization

This phase establishes the foundational parameters defining the administration scenario. This phase specifies four critical elements:

i. **Dosage Form and Formulation Characteristics:**

The starting point is explicit specification of what is being administered and in what physical state.

- *Drug substance properties:* Drug substance properties represent fixed constraints within QTAP and should be explicitly characterized because they strongly influence in-use performance. Properties such as solubility, pKa, lipophilicity, ionization, osmolality, viscosity, and permeability influence dissolution, tube adsorption, flow behaviour, compatibility with enteral nutrition, and the extent of dose delivery and absorption [36]. EFT feasibility assessment requires characterization beyond standard physicochemical properties. Wettability determines how readily a drug disperses in flush volumes. Hydrophobic drug particles may resist wetting and form aggregates irrespective of nominal solubility, increasing occlusion risk [40]. Polymorphic conversion induced by crushing can unpredictably alter solubility and stability [41]. They should be considered alongside formulation and administration factors when assessing route suitability and defining administration-related risks.
- *Dosage form classification:* Is the product a ready-to-use liquid (solution or suspension), a solid requiring manipulation (tablet to be crushed, capsule to be opened), or a product requiring reconstitution? This fundamental distinction profoundly affects subsequent attribute identification; ready-to-use suspensions have attributes related to suspension stability and viscosity [42], while crushed tablets introduce attributes related to crushing technique, particle size after crushing, and dose recovery from transfer steps [43].
- *Formulation properties:* Key characteristics include viscosity (affects flow through tubes), particle size distribution (affects blockage risk for suspensions and crushed tablets), pH (affects compatibility with tube materials and enteral formulas), osmolality (affects GI tolerance, particularly in neonates), and density/specific gravity (affects mixing and suspension characteristics) [14,44,45].
- *Intended versus off-label use.* It is critical to document whether the dosage form is designed and labelled for EFT administration or whether EFT use represents off-label manipulation. If a tablet formulation is labelled for intact oral swallowing but clinicians routinely crush it for tube administration, this off-label status elevates risk and warrants explicit documentation [46,47]. The manufacturer may not control crushing quality, yet QTAP can inform guidance or direct development of a purpose-designed alternative.

This dosage form characterization drives different administration quality priorities. The suspension requires focus on flow and blockage prevention, while the crushed tablet scenario requires focus on crushing technique, dose recovery, and appropriateness of the manipulation itself.

ii. **Feeding Tube Characteristics**

Feeding tubes represent the device interface that critically influences administration success. Characterization must address the range of tubes through which the product will be administered, as tube specifications directly determine flow feasibility and blockage risk.

- *Tube diameter range.* Feeding tubes span from 4 French (Fr) in extremely premature neonates to 24 Fr in adults. Each French unit equals 1/3 mm outer diameter. Flow resistance increases dramatically as tube diameter decreases, and blockage risk from particulates escalates in smaller tubes [36,48]. QTAP characterization must specify the range,; the product must be suitable for administration through 8 Fr to 18 Fr tubes, creating very different formulation requirements than those suitable for 14 Fr to 24 Fr tubes.
- *Tube materials.* Common materials include polyurethane (most common in modern tubes, relatively inert), silicone (softer, more flexible, used in long-term tubes), and polyvinyl chloride (PVC, older material, more prone to drug adsorption). Drug–tube material interactions vary. Some formulations adsorb significantly to PVC but not polyurethane, affecting dose recovery [49,50,51]. QTAP should specify which materials are relevant, such as those compatible with polyurethane and silicone nasogastric tubes.
- *Tube lengths and placements.* Tubes vary from ~30 cm for nasogastric tubes in neonates to >100 cm for nasojejunal tubes in adults. Longer tubes mean greater dead volume, requiring larger flush volumes for dose recovery, and longer transit time, increasing risk of formulation changes (settling, adhesion) during administration [10,52,53]. Tube placement affects whether formulation contacts gastric acid (NG, gastrostomy) or bypasses the stomach (NJ, jejunostomy), which can influence formulation stability and compatibility with enteral feeds [54].
- *Tube access points.* Some tubes have Y-ports or medication ports for administration; others require disconnecting feeding sets. The connection method affects spillage risk and ease of administration, influencing the practical feasibility of administration by caregivers.

iii. **User Population and Administration Setting**

Understanding who administers the product and in which setting it is used is essential for characterizing technique-dependent variability and for identifying administration attributes that may influence the probability of successful dose delivery.

- *User population stratification.* In hospital environments, nurses working in specialized units such as neonatal or pediatric intensive care typically receive extensive training in medication administration through fine-bore feeding tubes, have access to appropriate equipment (e.g., graduated syringes, crushing or dispersing devices), and operate within well-defined institutional protocols [55,56]. By contrast, practice in general wards is more variable due to less specialized training and equipment [57]. Pharmacy preparation benefits from controlled conditions but may introduce risks related to suspension stability and dose uniformity between preparation and administration [51]. Outside acute care, home caregivers (often parents or family members) frequently encounter constraints related to health literacy, language, emotional burden, and limited access to specialized devices. They rely predominantly on over-the-counter supplies and written instructions, leading to high variability in administration technique [58,59].
- *Setting characteristics.* The physical and organizational characteristics of the administration setting modulate these user-related factors. Controlled hospital settings generally provide dedicated preparation areas, access to sterile water for flushing, environmental control (temperature and cleanliness), availability of backup supplies in the event of errors or tube blockage, and oversight by experienced staff. In contrast, home administration typically occurs in kitchens or bedrooms, and may rely on tap water for flushing depending on local guidance. This is subject to variable ambient conditions, and offers limited redundancy if doses are wasted or tubes become occluded. Mobile or transport settings (e.g., ambulance or inter-facility transfer) impose additional constraints, including minimal preparation space, time pressure, and restricted capacity to troubleshoot tube blockages or administration failures.

iv. **Treatment Context and Clinical Constraints**

The broader therapeutic context determines which administration attributes become critical and which constraints must be actively managed within the QTAP.

- Dosing regimen characteristics such as frequent dosing, weight-based calculations or titration increases caregiver burden and the cumulative risk of measurement and preparation errors, particularly when small volumes are needed.
- Patient vulnerabilities add further constraint. Volume-restricted, immunocompromised, or fluid-sensitive patients face heightened risk around flush volumes, contamination and compatibility. Those receiving multiple medications through the same tube face additional complexity. Neonates in intensive care often present all these challenges simultaneously. Furthermore, pediatric patients possess unique and dynamically changing gastrointestinal environments, including age-dependent differences in gastric pH, gastrointestinal motility, enzyme activity, and absorptive capacity [60]. These physiological characteristics can influence medicine exposure and clinical response, making the control of administration-related attributes particularly important.
- Therapy duration affects the complexity of administration procedures. Short-term procedures may become unsustainable/risk-prone over chronic treatment [61].
- Concurrent enteral nutrition adds another layer of complexity. Many EFT-dependent patients receive continuous or bolus feeds that require careful coordination of medication timing, compatibility with enteral formulas, and potential feed-holding protocols, with implications for both drug exposure and nutritional status [62].
- Finally, the level of clinical monitoring determines how quickly administration errors are detected. In some settings, failures may go unnoticed until significant adverse clinical consequences emerge.

#### 3.1.2. Phase 2: Administration Attribute Identification

This phase systematically generates potential quality attributes relevant to the context characterized in phase 1. Attributes are organized across five categories:

i. **Dose Delivery Performance Attributes**

Dose delivery performance attributes address whether the intended dose reliably reaches the patient in the correct amount. For liquid formulations, key factors include measurement accuracy using oral syringes, dose losses due to tube adhesion or dead space, incomplete flushing, and consistency across repeated administrations and different users. For multi-dose presentations, dose accuracy must be maintained throughout the in-use period, accounting for evaporation, microbial growth, or excipient degradation [63]. For suspensions, uniformity and redispersibility are critical. The suspension must be easily homogenized by shaking and remain uniform throughout the administration window.

ii. **Flow Characteristics and Tube Compatibility Attributes**

Flow and compatibility attributes determine whether the formulation can pass through feeding tubes without resistance, blockage, or dose loss. Key considerations include tube flow time at typical administration rates. Excessive flow time burdens caregivers; very rapid flow may cause GI intolerance. Flow time depends on viscosity, tube diameter, and administration technique (gravity flow vs. gentle plunger pressure). The plunger force must also be assessed. Forces > 10 N exceed ideal ergonomic limits for handling tasks involving fingers and thus can fatigue caregivers, causing inconsistent delivery or incomplete dosing [64]. Tube blockage risk is driven by particle size, viscosity, administration rate, and flushing adequacy. Product adherence to tube materials can further reduce the effective delivered dose even when procedures are correctly followed [65]. Compatibility with enteral formulas should be evaluated. Short-term chemical stability during administration must be confirmed.

iii. **Preparation and Administration Process Attributes**

Preparation and process attributes capture the practical complexity and time required to prepare and administer the dose. This includes the number and sequence of discrete steps required, from shaking, crushing, or mixing through to connection, administration, and flushing, as well as the total procedure time. Ready-to-use liquids may take under 2 min, while crushed tablet preparation can take 8–10 min, creating significant burden for frequently administered medications.

Required equipment must also be considered. Specific oral syringes, crushing devices, mixing containers, and appropriate flush water may not always be available, particularly in home settings. For tablets, the crushing technique must be adequate, reproducibile, and practical. For suspensions and slurries, shaking or mixing instructions must be clear and effective. Administration rate and technique (gravity vs. plunger-driven, rapid vs. slow delivery) also affect feasibility. Flush protocols (flush volume, pre-flushing, and post-flushing) must be clearly defined and realistic for the intended user.

iv. **User Attributes**

User attributes focus on how users interact with the product, devices, and instructions, and whether these elements help prevent misuse. Clear, readable instructions are essential. They should be written at an appropriate health literacy level, highlight key steps, and include troubleshooting guidance. Visual cues are important. Users should be able to confirm adequate resuspension and read syringe graduations clearly when preparing and administering doses. Dosing devices must be easy to use and practical. They should fit bottle openings and tube ports and allow accurate measurement of small or fractional doses. Design features can also reactively reduce errors. Oral-only syringes that cannot connect to IV ports help prevent wrong-route errors [66]. Clear warnings against crushing modified-release formulations help prevent wrong-product errors [67]. Finally, training and visual aids support and facilitate safe use in real-world settings by helping users learn and demonstrate correct technique [68].

v. **Safety and Product Integrity Attributes**

Safety and product integrity attributes ensure that the administration process does not introduce additional risks or degrade product quality during in-use handling. For multi-dose containers, this includes the potential for microbial contamination during repeated access and the ability of the preservative system to maintain microbiological quality over the labelled in-use period [63]. For crushed tablets and powders, the risk of caregiver exposure to drug dust, particularly for cytotoxic, hormonal, or otherwise hazardous medicines, and the practicality of recommended protective measures must be considered [69,70]. Mitigation of cross-contamination when equipment is reused across multiple medications is a further critical attribute. Finally, in-use stability under real-world conditions, including repeated opening, ambient exposure, and storage between doses, must be assured. Packaging should be child-resistant but still practical for intended users (fast degradation–preparation just before administration).

Supplementary Table S1 provides an example attribute inventory for a ready-to-use oral suspension for pediatric EFT use. This list of 28 attributes provides comprehensive coverage of administration quality dimensions for this specific scenario.

#### 3.1.3. Phase 3: Critical Administration Attribute (CAA) Determination

This applies risk-based criticality assessment to the comprehensive attribute list generated in phase 2. Each attribute is evaluated against three criteria:

**Clinical impact** reflects the potential consequences of attribute variability on patient safety and therapeutic efficacy. It is defined as follows:

- High impact: Deviations could lead to serious adverse events, treatment failure, or clinically meaningful shifts in exposure, such as dose variability for narrow therapeutic index medicines or tube blockage preventing any dose delivery.
- Medium impact: Variation may cause minor adverse effects or reduced efficacy without serious harm.
- Low impact: Variation is unlikely to influence clinical outcomes, as with largely aesthetic features or non-consequential device characteristics.

**Variability risk** characterizes the likelihood that an attribute will deviate beyond acceptable limits under real-world conditions.

- High risk is associated with strong dependence on user technique, environment, or inherent product limitations (e.g., suspension uniformity heavily depending on caregiver shaking or dose recovery driven by flush volume).
- Medium risk is where some variation is expected but generally contained within acceptable bounds.
- Low risk is where the attribute is inherently stable or tightly controlled by design.

**The Human-Factor Impact** dimension captures how such variation translates into caregiver burden, feasibility of administration, adherence, and quality of life.

- High impact: Variability can compromise administration success, substantially increase workload, or cause distress (e.g., frequent tube blockages necessitating tube replacement).
- Medium impact: Variability generates inconvenience without fundamentally undermining adherence.
- Low impact: Variations are largely imperceptible or practically negligible.

**Figure 3:**
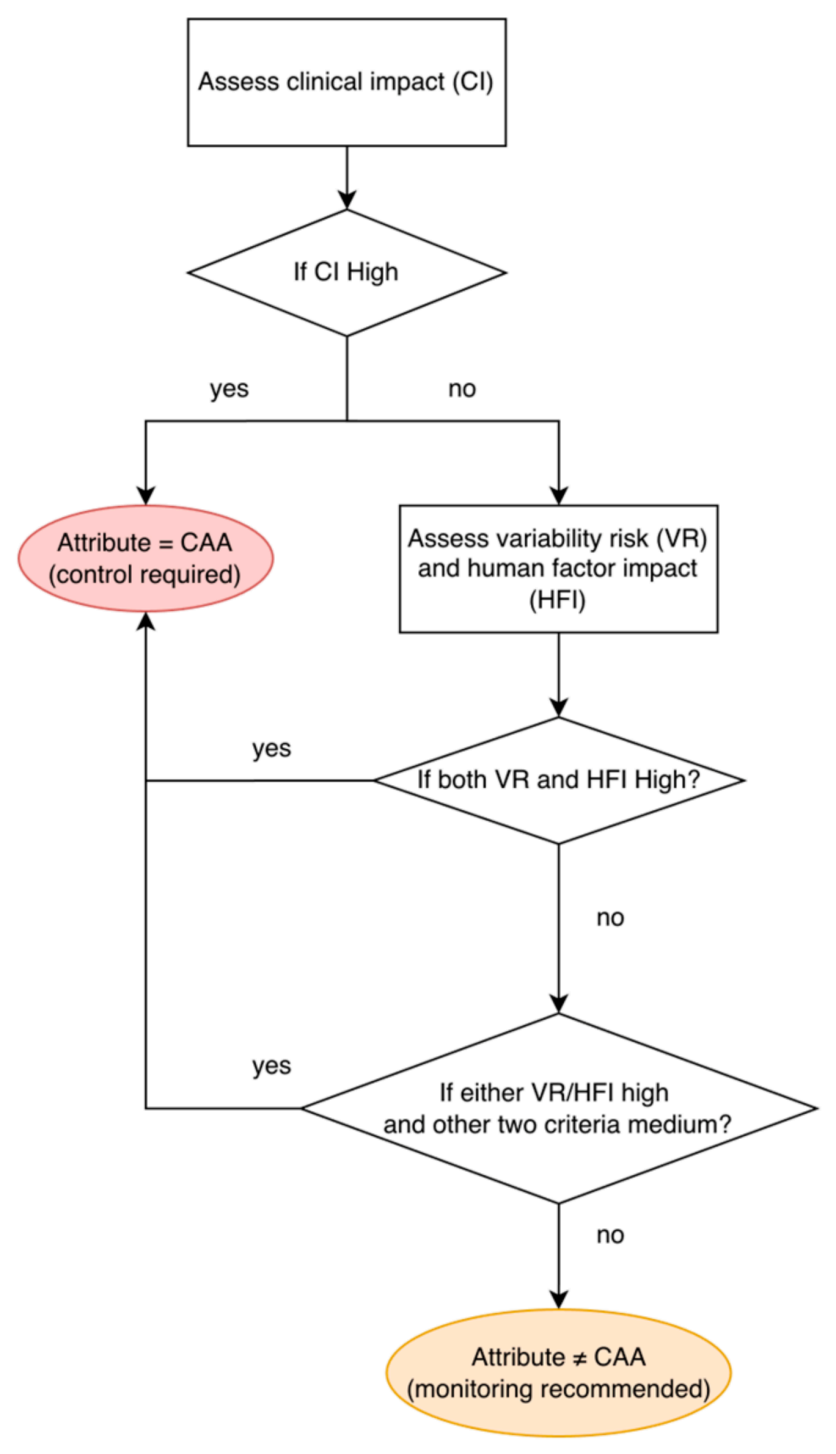
Flowchart illustrating CAA qualification based on assessment of three quality criteria (clinical impact (CI), variability risk (VR), and human-factor impact (HFI)).

Designation of a CAA is based on the combined profile across these three dimensions (Figure 3). An attribute qualifies as a CAA if it meets any of the following criteria: (a) High clinical impact regardless of other scores, (b) High variability risk combined with High HCP/patient impact, or (c) High on one criterion with Medium on both others. Application of this decision framework can be supported by the decision matrix as presented in Table 3. It yields a focused set of 5–8 CAAs from the initial 20–35 attribute list. It helps with transparency and consistency across products and project teams. The criticality characterizations can be revisited iteratively as emerging data from formulation development, in-use testing, and clinical experience, refining the understanding of each attribute’s behaviour and consequence profile.

After identifying attributes that qualify as CAAs, it is important to avoid assuming that all remaining attributes are inherently risk-free and of negligible relevance. Attributes classified within the orange categories (Table 3) may still present a low to moderate level of risk to administration performance, even though they are not deemed critical. These attributes warrant careful consideration and monitoring to ensure they do not cumulatively impact product and clinical outcomes.

## 4. Application of QTAP to Enteral Feeding Tube Scenarios

To demonstrate QTAP framework application, the 3-phase methodology was applied to three scenarios representing the spectrum of EFT administration complexity: (1) a ready-to-use oral suspension, (2) tablets requiring off-label crushing for tube administration, and (3) a low-viscosity oral solution. Each scenario is assessed through systematic Phase 1–3 of the framework.

### 4.1. Phase 1: Administration Context Characterization

Table 4 summarizes the administration contexts for the three scenarios, highlighting critical differences that drive subsequent attribute identification and criticality assessment.

- Scenario 1 (suspension) must address formulation-controlled attributes (uniformity, particle size) to ensure consistent dose delivery through fine bore tubes.
- Scenario 2 (crushed tablet) highlights a fundamental problem. Crushing creates quality risks such as variable particle size, incomplete dose recovery, and inconsistent technique that the manufacturer cannot control.
- Scenario 3 (solution) has simpler flow characteristics but faces the challenge of precise dosing for narrow therapeutic index drugs in fluid-restricted neonates where measurement errors, drug adsorption to feeding tube materials and flush volume directly impact both efficacy and safety.

The tube diameter ranges differ significantly:

- Scenario 1 must accommodate the pediatric tube (8 Fr).
- Scenario 2 uses larger gastrostomy tubes, reducing flow constraints.
- Scenario 3 spans the full range, including extremely small neonatal tubes (6 Fr).

User populations present distinct capability profiles. NICU nurses (Scenario 3) possess specialized training and operate in controlled environments with backup support. Pediatric ward nurses (Scenario 1) have moderate EFT experience and institutional protocols. Home caregivers (Scenarios 1 and 2) represent the highest variability, ranging from highly engaged parents managing complex medical care to overwhelmed caregivers with limited health literacy. This user variability directly influences which administration attributes become critical based on technique dependency.

### 4.2. Phase 2: Administration Attribute Identification

For each scenario, the administration attributes across five QTAP categories were systematically identified, informed on the literature-, guidance-, and expert judgement-based approach described in Section 3.1. The comprehensive attributes (provided in Supplementary Material Table S1) ranged from 18 attributes for the solution (simplest scenario with minimal preparation) to 32 attributes for the crushed tablet (most complex with multi-step manipulation). Table 5 presents a condensed comparison showing representative attributes in each category, illustrating how dosage form drives attribute relevance.

The ready-to-use formulations (suspension, solution) generate attributes predominantly controlled by formulation design (viscosity, particle size, osmolality, and stability), where the manufacture determines quality through formulation composition and manufacturing process control. The crushed tablet scenario introduces attributes (crushing adequacy, transfer losses) inherently dependent on user technique and thus higher variability risk.

### 4.3. Phase 3: Critical Administration Attribute Determination

Each identified attribute was systematic criticality assessed using the three-criterion risk assessment framework: clinical impact, variability risk, and healthcare professional/patient impact (Section 3.1.3). Attribute scoring was performed in accordance with the decision matrix (Table 3), which systematically defines when an administration attribute meets CAA criteria. The resulting criticality assessments and risk classifications are presented in Table 6.

The criticality assessment identified 5–6 CAAs per scenario from an initial list of 18–32, helping focus development efforts where they matter most. Notably, for Scenario 2 (crushed tablet), the appropriateness of crushing was identified as a CAA, but the appropriate control strategy is not to improve the crushing process. Instead, crushing should be contraindicated and an alternative formulation developed. This illustrates how QTAP can reveal that certain administration approaches are fundamentally incompatible with quality objectives.

### 4.4. CAA Profiles Across Dosage Forms

Table 7 summarizes the final CAA determinations across all three scenarios, revealing how administration quality priorities shift dramatically with dosage form despite serving the same therapeutic purpose (delivering drugs via EFT to pediatric patients).

This comparative analysis reveals a critical insight. The dosage form selection profoundly impacts administration quality risk profile. Liquid formulations (suspension, solution) enable manufacturer control of most CAAs through formulation design, while the crushed tablet approach transfers quality responsibility to end users who lack the tools, training, or standardized processes to ensure consistent outcomes. From a QbD perspective, crushing tablets not designed for crushing represents a systematic quality compromise. Multiple CAAs (crushing adequacy, dose recovery, and powder exposure) cannot be adequately controlled through manufacturer specifications or instructions alone.

## 5. Discussion

This work proposes the QTAP as an extension of established QbD and QTPP principles. It aims to address a gap of a structured approach to administration quality. While conventional QbD frameworks effectively translate patient needs into QTPP elements and subsequently into CQAs, CMAs and CPPs, the administration step is typically treated as an operational consideration rather than a design variable. In practice, for medicines administered via EFTs, the route of administration is usually captured as a single QTPP line item (e.g., oral and via feeding tube), while the detailed mechanics of dose delivery, tube compatibility and user interaction are left to late-stage feasibility work or local guidelines. The QTAP framework addresses this gap by explicitly defining the administration quality domain (dose delivery, flow, preparation, user interface and safety) and linking it to CAAs that can be integrated into the existing QbD workflow. The application of QTAP to three EFT scenarios demonstrates that administration quality can be systematically assessed, prioritized, and integrated into pharmaceutical development using established QbD principles.

### 5.1. QTAP in the Context of the EFT Administration Literature and Practice

There is little guidance on what constitutes successful administration in measurable terms. Recent reviews and observational studies converge on three findings: EFT administration is associated with frequent dose loss and tube obstruction, practice is highly variable, and many problems arise when products are manipulated [8,71,72]. These studies identify failure points, such as inadequate flushing or particle size-related blockage, but do not translate these into prospective design requirements. Karkossa et al. showed that delivered dose for pediatric ibuprofen suspensions depends not only on formulation factors but also on tube characteristics and the ratio of total administered volume to tube volume, directly linking operational choices to exposure [8]. Other authors have mapped the administration process into flow charts to highlight critical points at which drug loss or interaction with feeds occurs. Quality improvement programmes in intensive care and long-term care settings demonstrate that structured interventions checklists, pharmacist-led education, and double checks can substantially reduce errors and feeding tube obstructions, indicating operation at the level of practice improvement rather than product design [73,74]. These findings reveal that administration failures are (i) common and multifactorial and (ii) amenable to systematic analysis, but (iii) typically addressed reactively through local practice improvements rather than proactively embedded in product design. Current documents such as the Handbook of Drug Administration via Enteral Feeding Tubes categorize products based on empirical experience (suitable, caution, and not suitable), but these are descriptive compilations of observed outcomes, not prospective design frameworks [18]. They document what has happened, not what should be designed. Moreover, they rely on limited or anecdotal evidence, with recommendations often based on small studies or theoretical concerns rather than systematic testing under conditions representative of clinical practice. The QTAP framework represents a shift from reactive documentation to proactive design. Rather than discovering administration problems through clinical use and retrospectively attempting mitigation, QTAP identifies potential quality attributes during development, assesses their criticality, and establishes control strategies before clinical exposure. This parallels the evolution that occurred in manufacturing quality with QbD implementation where quality-by-testing had evolved to quality-by-design.

### 5.2. Integration with QbD, Design Space and Feasibility Testing

Within ICH Q8, a design space is defined as the multidimensional region of material attributes and process parameters that has been demonstrated to assure quality [29,31]. QTAP-identified CAAs do not create a separate design space. Instead, QTAP functions upstream of design space definition, analogous to how TPP informs QTPP. The three-phase QTAP process, including context characterization, attribute identification, and criticality assessment, culminates in a set of CAAs that should be treated in the same way as CQAs. For example, if dose recovery through 8 Fr tubes and blockage incidence under specified flush protocols are identified as CAAs, then formulation CMAs (e.g., viscosity, particle size, and wetting properties) and process parameters (e.g., milling, suspension structuring) become attributes for an administration-relevant design space, rather than focusing solely on conventional metrics such as assay and release. In practical terms, QTAP informs which administration endpoints are built into DoE studies and in vitro EFT simulations recommended by regulators. This does not create a separate administration design space but enriches the existing design space with additional response variables and constraints that reflect real-world administration.

QTAP also reframes feasibility testing. Current practice around EFT administration is dominated by small feasibility studies, observational work on obstruction and error rates, and implementation projects introducing guidance or training [7,75,76,77]. These efforts consistently show benefit but are often reactive, driven by post-marketing problems. QTAP does not replace such feasibility work; rather, it provides a framework to systematize the feasibility study. It clarifies what feasibility questions must be answered (e.g., which tube sizes) and ties those questions to predefined CAAs. A critical point is that QTAP alone cannot guarantee successful EFT administration: robust in vitro and, where appropriate, clinical or simulation-based studies remain essential. QTAP’s value lies in making these activities more structured, better justified, and more comparable across products. The case examples in this paper illustrate that administration challenges are dosage form-specific; ready-to-use suspensions, crushed tablets and concentrated solutions exhibit different dominant risks, supporting the argument that generic checklists are insufficient and that a structured, context-specific approach is needed. Phase 1 and Phase 2 specify which administration scenarios and endpoints matter most for a given product, so feasibility and in vitro studies can be targeted to those predefined questions. This makes the resulting data easier to interpret, compare and justify in submissions.

### 5.3. Benefits and Potential Drawbacks of a Systematic QTAP Approach

From an industrial perspective, the most tangible benefit of QTAP is risk reduction around late-stage and post-marketing problems related to administration [32]. The criticality assessment framework identifies high-risk attributes early when formulation modifications are feasible and relatively low-cost. For example, recognizing that tube blockage is a CAA for 8 Fr pediatric tubes establishes a particle size target (d90 < 15–20 μm) that influences milling process selection during formulation development. Addressing this proactively through formulation design prevents the scenario where a product meets all traditional specifications yet fails in clinical use due to tube blockages, requiring post-approval formulation changes or restricted labelling. FDA guidance on oral products administered via EFTs already expects sponsors to consider in vitro tube testing, compatibility with enteral feeds and practical in-use conditions [23]. EMA guidance explicitly requires demonstration of EFT administration feasibility, including tube blockage risk assessment and dose recovery [78]. QTAP provides the structured framework for generating this evidence systematically. A structured QTAP, embedded early in development, can help sponsors anticipate these expectations, identify high-risk administration scenarios and design focused studies, for example, increasing viscosity to improve suspension stability but potentially compromising tube flow. If tube flow is a CAA but suspension stability is not (because adequate resuspension is achievable with reasonable shaking), formulation optimization prioritizes maintaining viscosity within tube-compatible ranges. Rather than generic claims that a product is suitable for tube administration, QTAP-guided development enables specific, data-supported statements: “Tube blockage rate < 1% demonstrated in simulated administration through 8 Fr tubes (*n* = 50 administrations); dose recovery 96 ± 2% achieved with 5 mL flush protocol validated with intended users”. This specificity addresses regulatory expectations while providing clinicians with actionable information for practice. In addition, QTAP makes the rationale for product labelling, in-use instructions and any restrictions on tube size or feeding regimens traceable to an explicit administration risk assessment, which may support clearer regulatory dialogue and more transparent submissions. For hospitals and long-term care providers, QTAP-informed products can translate into fewer tube obstructions, more realistic instructions, and better alignment between product requirements and real-world workflows.

A matter of concern could be whether QTAP simply adds another layer of complexity on top of QTPP, CQAs and existing risk management activities. The literature shows that tools, per se, are not the limiting factor; rather, the problem is fragmentation between development, regulatory and clinical perspectives. QbD concepts are typically applied to manufacturing quality, while EFT initiatives focus on bedside practice, with little explicit linkage [37,73,74]. The intention of QTAP is therefore not to proliferate frameworks but to provide a connecting lens. If implemented as a standalone checklist, it would indeed add burden. However, if embedded into existing structures, it mainly reorganizes information that development teams and clinical stakeholders already hold. The outputs (CAAs and contextual descriptions) feed directly into the same risk assessment matrices used for CQAs, inform the same design space mapping, and ultimately shape the same control strategy. QTAP reorders and sharpens thinking about administration but does not introduce new regulatory constructs beyond those already present in QbD. The complexity lies mainly in the initial cultural shift recognizing administration as a quality concern, rather than in additional regulatory requirements.

### 5.4. Integrative Value for Regulators, Industry and Healthcare Providers

The value of QTAP becomes most evident when considering how industry, regulators and healthcare providers interact around EFT medicines. The industry needs data-supported instructions to mitigate the risks of off-label manipulation and to extend consideration to neonatal and pediatric populations [14]. However, the decision process for which scenarios to test, which endpoints to prioritize, and how to connect in vitro findings back to formulation and process variables is often implicit and product-specific. Regulatory agencies have begun to articulate expectations for EFT-relevant data [23,78]. FDA guidance on oral products administered via EFT calls for risk-based in vitro testing to support product-specific labelling, with recommendations on tube selection, dispersion methods, media and evaluation of dose recovery and blockage risk [23]. Clinical and professional bodies, in parallel, have produced handbooks, checklists and practice recommendations intended to reduce errors and tube complications in hospitals and long-term care [18,79]. Yet the decision logic linking clinical need, study design and final labelling often remains implicit. QTAP offers a way to make that logic explicit. By starting from a systematic mapping of administration attributes and ranking them for clinical impact, variability risk and user impact, the framework helps sponsors justify the selection of tube sizes, feed types, and administration conditions included in in vitro EFT studies. In addition, it justifies why specific metrics (e.g., minimum acceptable dose recovery, maximum blockage incidence) were chosen, and how these are reflected in the label and instructions for use. For regulators, this provides a clear line of sight from patient needs to product design and in-use performance. The FDA’s Patient-Focused Drug Development (PFDD) initiative prioritizes incorporating patient and caregiver perspectives throughout development, including administration convenience, treatment burden, and practical usability [80]. QTAP operationalizes these patient-focused considerations within a quality framework, translating patient needs (e.g., minimizing preparation time) into measurable quality attributes subject to risk assessment and control [78]. The EMA’s Q&A on pediatric EFT products explicitly requires developers to provide data on tube blockage risk, dose recovery, and administration ease [22]. These elements emerge naturally from QTAP Phase 2 attribute identification, thus helping to systematically address the regulatory expectations through a structured framework. Human-factor engineering requirements, particularly for combination products, intersect with QTAP. FDA guidance on applying human factors to medical devices requires demonstrating that users can safely and effectively operate products, including identifying and mitigating use-related risks [23]. QTAP provides the quality framework that human-factor validation studies must demonstrate. For products requiring human-factor validation, QTAP clarifies what success criteria should be evaluated: achieving adequate suspension uniformity through shaking (if that is a CAA), measuring accurate doses within acceptable error limits, and executing flush protocols achieving target dose recovery. For hospitals and long-term care facilities, QTAP-informed products may better match real-world practice constraints. Because QTAP explicitly incorporates user populations, settings and training infrastructure, it offers a mechanism for these real-world constraints to influence development decisions, rather than being discovered only after product launch.

### 5.5. Limitations and Future Directions

This manuscript introduces the QTAP concept and demonstrates its application to three representative scenarios, but does not provide comprehensive validation. The scenarios were assessed by the authors based on literature review, clinical practice knowledge, and formulation science principles, but systematic user testing with caregivers and healthcare professionals was not conducted to empirically verify attribute inventories or criticality assessments. Future work should include prospective validation where QTAP-identified CAAs are tested through human-factor studies, simulated-use testing, and ultimately real-world monitoring to confirm that attributes designated as critical indeed predict administration quality failures when not controlled. The framework is presented in the context of EFT administration for high-resource settings. Administration practices, user populations, and available resources differ globally. Home caregivers in low-resource settings may lack access to clean water for flushing, sterile equipment, or specialized administration devices, creating different attribute inventories and criticality profiles. QTAP should be adapted to regional context when products are developed for global markets.

Several research opportunities emerge from this initial QTAP framework development. Validation studies applying QTAP to products and comparing QTAP-guided development outcomes with traditional approaches would strengthen evidence for the framework’s value. Standardization of attribute inventories for common routes and dosage forms would facilitate QTAP implementation [29,30,31,32]. Rather than each development team independently generating Phase 2 attributes, industry consortia or regulatory bodies could develop reference attribute libraries for EFT liquids, EFT crushed solids, and other common scenarios. Developers would customize these templates to their specific products, improving consistency and reducing duplication of effort. Although the proposed QTAP framework captures the principal administration-related domains, it should be recognized that additional attributes may also become relevant depending on the product and clinical context. These include compatibility with food vehicles, co-administration with enteral nutrition, the influence of meal timing on administration performance, and other route-specific practical constraints that may affect dose delivery and user acceptability. Inhalation products present similar challenges. The quality product in the device must successfully deliver a respirable dose to the lungs, depending on user inhalation technique, device–patient interface, and environmental factors (humidity, temperature). Topical products (e.g., ophthalmic drops, otic solutions, and nasal sprays) have administration quality attributes related to dropper design, bottle orientation, and dose delivery consistency. The framework’s structure (characterize context, identify attributes, and assess criticality) provides a generalizable approach adaptable to certain routes where administration involves patient/caregiver action beyond simply swallowing an intact solid. However, it may have limited applicability to novel administration routes or delivery technologies (e.g., long-acting injectables, novel inhalation devices, and digital health-enabled systems) where less clinical experience exists to guide Phase 2 attribute identification or Phase 3 criticality scoring. Successful implementation would require gathering user insights, conducting formative human-factor research, and iterating as understanding of administration challenges develops [81,82]. The growing availability of machine learning and AI-assisted modelling tools presents an opportunity to accelerate and support this process. By integrating experimentally derived datasets with predictive modelling approaches, QTAP could evolve into a dynamic and data-driven system capable of forecasting administration performance by identifying high-risk attribute combinations and proposing instruction-based interventions. AI/ML-coupled QTAP therefore represents a promising future direction for decision-making and advancing administration-quality design.

## 6. Conclusions

Medications administered via EFTs present unique pharmaceutical and clinical complexities that extend beyond formulation design. In this study, application of the QTAP framework to three EFT scenarios demonstrates that administration quality can be systematically characterized, assessed and prioritized through a risk-based approach. The framework enabled the identification of a focused set of CAAs that can be used to guide formulation development, testing strategies, and administration instructions. The analysis further showed that administration quality requirements vary across administration scenarios and are influenced by multiple interacting factors, including dosage form, patient population, clinical setting, user characteristics, and feeding tube specifications. As a conceptual extension of existing QbD principles, QTAP is intended to complement rather than replace current approaches such as QTPP. Future prospective validation, including human-factor studies, in vitro investigations, and real-world evaluations, is needed to establish its practical utility and predictive value. If validated, QTAP could support more patient-centred product development and facilitate the proactive incorporation of administration quality into pharmaceutical design.

## Figures and Tables

**Table 1:** Non-exhaustive list of QTAP attribute examples.

Attribute Category	Example Attributes
Formulation flow characteristics	Viscosity, particle size distribution, suspension stability, and rheological properties that govern flow through tubes of varying diameters and lengths
Dose delivery performance	Accuracy of dose measurement, reproducibility, completeness of dose delivery, consistency across multiple administrations
Tube compatibility	Compatibility with tube materials (polyurethane (PU), silicone, polyvinylchloride (PVC)) regarding drug adsorption, absence of precipitation or phase separation during administration, prevention of tube blockage
Administration performance requirements	Preparation (*complexity and number of steps required such as shaking, crushing, mixing, drawing, and flushing*), time required, device needs, opportunity for errors, flush requirements: volume of flush needed to achieve complete dose delivery, appropriateness for volume-restricted patient populations, standardization across tube types
In-use product behaviour	Stability during administration, maintenance of product integrity throughout the use period. EFT-specific considerations include whether suspensions remain uniform during the administration window, and whether products tolerate brief room temperature exposure during preparation.
Healthcare professional factors	Technique dependencies (*draw accurate doses into oral syringes, connect to tube ports without spillage, detect resistance during administration, and recognize when dose delivery is complete*), training requirements, clarity of instructions, caregiver burden
Patient-centric factors	Physical accessibility, adherence enablers, treatment burden, and quality-of-life considerations

**Table 2:** The TPP → QTPP → QTAP Continuum.

Framework	Focus	Time Horizon	Primary Question	Example Element	Controlled By
TPP	Clinical intent	Treatment duration	What therapeutic outcome?	Efficacy in pediatric infection	Clinical design, drug properties
QTPP	Product quality	Shelf life (2+ years)	What product attributes enable outcome?	Drug content 95–105%, particle size controlled	Manufacturer (formulation, process)
QTAP	Administration quality	Administration event (minutes)	What administration attributes ensure dose delivery?	Tube flow < 2 min, blockage rate < 1%, dose recovery > 95%	Formulation design + HCP technique

**Table 3:** Example of a CAA decision matrix illustrating the classification of administration attributes as CAA or non-CAA based on qualitative scores for CI, VR, and HFI. Colour coding: Red = high risk; yellow = medium risk; green = low risk.

Attribute	Clinical Impact (CI)	Human-Factor Impact (HFI)	Variability Risk (VR)	CAA?	Justification
Attribute A	High	High	High	YES	Essential control when all scores high
Attribute B	High	Medium/Low	Medium/Low	YES	High CI indicates automatic CAA designation
Attribute C	Medium/Low	High	High	YES	Both HFI and VR high-risk factors
Attribute D	Medium	Medium	High	YES	One high (HFI or VR) and two medium criteria
Attribute E	Low	Medium	High	NO	Does not meet CAA threshold but monitoring recommended
Attribute F	Medium	Medium	Medium	NO	Does not meet CAA threshold but monitoring recommended
Attribute G	Low	Low	Low	NO	Low risk non-CAA

**Table 4:** Administration context characterization for three EFT scenarios.

Parameter	Scenario 1: Oral Suspension	Scenario 2: Crushed Tablet	Scenario 3: Oral Solution
**Product and Indication**	Antibiotic 200 mg/5 mL; bacterial infections	Immunosuppressant 50 mg tablet; transplant maintenance	Antiepileptic 50 mg/mL; seizure control
**Dosage Form**	Ready-to-use aqueous suspension	IR tablet, film-coated; off-label crushing	Ready-to-use aqueous solution
**Physical Properties**	Viscosity 80–120 cP; d90 < 20 μm; pH 5.5–6.5; osmolality ~300 mOsm/kg	Hardness 8–12 kp; 6 mm diameter; film coating	Viscosity < 5 cP; pH 4.0–5.0; 280 mOsm/kg
**Feeding Tube Range**	8–14 Fr NG tubes (PU); 30–50 cm length	14–18 Fr gastrostomy (silicone)	6–18 Fr NG/G-tubes (all materials); variable lengths
**Patient Population**	Hospitalized children; 1 month–12 years; acute infection	Pediatric transplant; recipients 2–16 years; chronic therapy	Neonates–infants; NICU/narrow therapeutic index
**Primary Users**	Hospital nurses (ward); parent caregivers (home)	Home caregivers (parents); hospital pharmacists	NICU nurses; specialized training
**Setting**	Hospital wards; supervised home use	Predominantly home (pediatric transplant); unsupervised	NICU; controlled hospital environment; high supervision
**Dosing Regimen/characteristics**	Weight-based, TID × 7–10 days	Fixed dose, BID (chronic therapy); narrow therapeutic index; monitoring	Weight-based, TID-QID
**Clinical Constraints**	Moderate therapeutic window	Narrow therapeutic index; immunosuppression monitoring	Very narrow therapeutic index; fluid restrictions in neonates
**Off-label Status**	Labelled for EFT use	Not labelled; crushing contraindicated in some formularies	Labelled for EFT use

**Table 5:** Representative administration attributes by scenario.

Attribute Category	Suspension	Crushed Tablet	Solution
**Dose Delivery**	Suspension uniformity after shaking Dose measurement accuracy Dose recovery (% delivered) from tube	Crushing completeness (fragment size) Dose recovery from crushing device; dose recovery from mixing container; dose recovery from transfer steps Particle suspension stability Dose precision across caregivers	Dose measurement accuracy (small volumes) Dose completeness (minimal residual)
**Flow/Tube Compatibility**	Tube blockage risk (particles) Flow time through 8–14 Fr tubes Adsorption to polyurethane Plunger force requirement Compatibility with enteral formulas	Tube blockage risk (crushed particles, very high) Slurry viscosity variability Particle settling during administration Compatibility with residual feeds	Rapid flow characteristics (low viscosity) Osmolality for GI tolerance Compatibility with tube materials Minimal adherence
**Preparation and process**	Shaking requirements (inversions, vigour) Flush volume needed (dose recovery) Total administration time Equipment: oral syringe, flush vehicle	Crushing method standardization/equipment variability (mortar pestle, devices) Transfer steps (3–4 opportunities for loss) Total time (~8–10 min; high burden) Flush volume (higher due to adherence)	Minimal preparation Flush volume (critical in fluid restricted) Time (~1–2 min) Equipment: precision syringe
**User Interface**	Instructions for use (shaking, flushing) Visual confirmation of uniformity Dosing syringe graduation readability	Crushing adequacy assessment (no clear endpoint) Multi-step error potential (cumulative) Technique-dependent outcomes IFU for crushing (often missing)	Measurement precision for small volumes Concentration error prevention Syringe selection (0.1 mL graduations) Visual clarity of solution
**Safety**	Microbial stability (multi-dose) Contamination during withdrawal Child-resistant closure In-use stability (30 days)	Powder exposure during crushing Cross-contamination (reused equipment) Appropriateness of crushing (formulation suitability)	Accidental concentration errors Hyperosmolar complications In-use stability

**Table 6:** The criticality assessment results for selected high-priority attributes from each scenario, demonstrating the decision logic. Colour coding: Red = high risk; yellow = medium risk; green = low risk.

Scenario	Attribute	Clinical Impact	Variability Risk	HCP/Patient Impact	CAA?	Rationale
**Suspension**	Suspension uniformity	High (dose ± 20–30% from settling)	High (shaking technique varies)	Medium (not visible to user)	**YES**	H+H combination; formulation must minimize settling
	Tube blockage risk	High (zero dose delivery)	High (particle size, tube diameter)	High (traumatic, costly)	**YES**	H+H+H; drives particle size specification
	Dose recovery	High (dose losses affect outcomes)	High (flush technique varies)	High (fluid-restricted patients)	**YES**	H+H+H; requires validated flush protocol
	Child-resistant cap	High (poisoning risk)	Low (standard testing)	Medium (some difficulty opening)	**YES**	Standard packaging requirement, not unique CAA
**Crushed Tablet**	Crushing completeness	High (fragments block tubes)	High (technique, equipment vary)	High (time, physical demand)	**YES**	H+H+H. but uncontrollable by manufacturer
	Dose recovery	High (15–30% losses reported)	High (transfer steps, adherence)	High (dose uncertainty)	**YES**	H+H+H; systematic quality compromise
	Appropriateness of crushing	High (loss of formulation features)	Low (consistent if done)	High (off-label creates liability)	YES *	H+H; largely depends on user execution
	Powder exposure	High (for hazardous drugs)	Medium (technique-dependent)	High (caregiver safety concern)	**YES**	For cytotoxics/teratogens; may contraindicate crushing
**Solution**	Dose measurement accuracy	High (narrow TI, weight-based)	High (small volumes, concentrated)	Medium (requires precision)	**YES**	H+H; concentrated solutions amplify measurement errors
	Flush volume	Medium (dose losses)	Medium (technique)	High (fluid restrictions in neonates)	**YES**	M+M+H combination; clinical constraint drives CAA
	Osmolality	High (GI intolerance, rapid admin)	Low (formulation controlled)	High (feeding intolerance)	**YES**	H+H despite low variability; specification critical

* May indicate crushing contraindicated; requires alternative formulation.

**Table 7:** Summary of confirmed CAAs across dosage forms.

Scenario	Confirmed CAAs (*n* = 5–6)	Primary Quality Risk Drivers	Overall Administration Quality Risk
**Suspension (RTU)**	1. Suspension uniformity 2. Tube blockage prevention 3. Dose recovery from tube 4. Flush volume requirements 5. Administration instructions 6. Child-resistant cap	• Formulation stability (settling) • Particle size control • User shaking technique • Flush protocol adherence	Moderate—Controllable by manufacturer through formulation design; technique dependencies manageable with clear instructions
**Crushed Tablet**	1. Crushing completeness 2. Dose recovery (cumulative losses) 3. Appropriateness of crushing 4. Tube blockage risk 5. Powder exposure (hazardous drugs) 6. Administration instructions	• User crushing technique • Equipment variability • Formulation suitability • Multiple transfer steps • Systematic dose losses	High—Largely uncontrollable by manufacturer; quality dependent on user execution; may contraindicate crushing entirely
**Solution**	1. Dose measurement accuracy 2. Flush volume (fluid restrictions) 3. Osmolality 4. Concentration error prevention 5. Compatibility across tube types	• Measurement precision requirements • Concentrated formulation risks • Fluid management constraints • Neonatal GI sensitivity	Low—Moderate Controllable through formulation design and dosing device selection; minimal technique dependency

## Data Availability

No new data is generated in this study.

## Supplementary Materials

The following supporting information can be downloaded at: https://www.mdpi.com/article/10.3390/pharmaceutics18091089/s1, Table S1: Comprehensive Administration Attributes for Three EFT Scenarios.

## Abbreviations

The following abbreviations are used in this manuscript:

AI	Artificial Intelligence
BID	Bis In Die; Two Times a Day
CAAs	Critical Administration Attributes
CI	Clinical Impact
CPPs	Critical Process Parameters
CQAs	Critical Quality Attributes
EFTs	Enteral Feeding Tubes
EMA	European Medicines Agency
FDA	US Federal Drug Administration
Fr	French size
G (tube)	Gastrostomy tube
GI	Gastrointestinal
HFI	Human-Factor Impact
ICH	International Council for Harmonization of Technical Requirements for Pharmaceutical for Human Use
ML	Machine Learning
NG	Nasogastric
NICU	Neonatal Intensive Care Units
NJ	Nasojejunal
PFDD	Patient-Focused Drug Development
PU	Polyurethane
PVC	Polyvinylchloride
QbD	Quality by Design
QID	Quarter In Die; Four Times a Day
QTPP	Quality Target Product Profile
TID	Ter In Die; Three Times a Day
TPP	Target Product Profile
VR	Variability Risk

## References

[1] Smith T., Micklewright A., Hirst A., Stratton R., Baxter J. (2011). Artificial Nutrition Support in the UK 2000–2010: A Report by the British Artificial Nutrition Survey (BANS), a Committee of BAPEN (The British Association for Parenteral and Enteral Nutrition).

[2] Burdall O.C., Howarth L.J., Sharrard A., Lee A.C.H. (2017). Paediatric enteral tube feeding. Paediatr. Child Health.

[3] Paulsson M., Hamre Svendsen R., Andersen J.K.N., Kälvemark Sporrong S., Andersson Y., Tho I. (2025). Challenges and Considerations in Manipulating Oral Dosage Forms in Paediatric Healthcare Settings: A Narrative Review. Acta Paediatr..

[4] Zahn J., Hoerning A., Trollmann R., Rascher W., Neubert A. (2020). Manipulation of Medicinal Products for Oral Administration to Paediatric Patients at a German University Hospital: An Observational Study. Pharmaceutics.

[5] Pancorbo-Hidalgo P.L., García-Fernandez F.P., Ramírez-Pérez C. (2001). Complications associated with enteral nutrition by nasogastric tube in an internal medicine unit. J. Clin. Nurs..

[6] de Villiers M.M., Vogel L., Bogenschutz M.C., Fingerhut B.J., D’Silva J.B., Moore A. (2010). Compounding rifampin suspensions with improved injectability for nasogastric enteral feeding tube administration. Int. J. Pharm. Compd..

[7] Reindel K., Zhao F., Hughes S., Dave V.S. (2017). In Vitro Evaluation of Eslicarbazepine Delivery via Enteral Feeding Tubes. Hosp. Pharm..

[8] Karkossa F., Lehmann N., Klein S. (2022). A systematic approach for assessing the suitability of enteral feeding tubes for the administration of controlled-release pellet formulations. Int. J. Pharm..

[9] Hoover A., Chitranshi P., Momot M., Tyner K., Wokovich A. (2021). In vitro evaluation of enteral tube administration of lansoprazole orally disintegrating tablets. Pharm. Dev. Technol..

[10] Klang M.G. (2023). Developing guidance for feeding tube administration of oral medications. J. Parenter. Enter. Nutr..

[11] Sripongpun P., Lertpipopmetha K., Chamroonkul N., Kongkamol C. (2021). Diarrhea in tube-fed hospitalized patients: Feeding formula is not the most common cause. J. Gastroenterol. Hepatol..

[12] Ruzsíková A., Součková L., Suk P., Opatřilová R., Kejdušová M., Šrámek V. (2015). Quantitative analysis of drug losses administered via nasogastric tube—In vitro study. Int. J. Pharm..

[13] Thong M.Y., Manrique Y.J., Steadman K.J. (2018). Drug loss while crushing tablets: Comparison of 24 tablet crushing devices. PLoS ONE.

[14] Boullata J.I. (2009). Drug administration through an enteral feeding tube. AJN Am. J. Nurs..

[15] Williams N.T. (2008). Medication administration through enteral feeding tubes. Am. J. Health-Syst. Pharm..

[16] Tillott H., Barrett D., Ruan J., Li V., Merrick S., Steed H., Morrissey H., Anthony Ball P. (2020). Survey of nurses’ knowledge and practice regarding medication administration using enteral tubes. J. Clin. Nurs..

[17] Watson C., Kerr S., Davies J., Stirling H., Webb E., Batchelor H. (2018). G219 Hydrocortisone tablets: Human factors in manipulation and their impact on dosing accuracy. Arch. Dis. Child..

[18] Phillips M.S. (2007). Handbook of Drug Administration via Enteral Feeding Tubes. Am. J. Pharm. Educ..

[19] Wright D.G.R., Merriman H., Smithard D., Smyth J., Welsh N. (2021). Medication Management of Patients with NG, PEG or Other Feeding Tubes.

[20] Ayhan Y.E., Özkanlı Ö.F., Gözelizmir Ş., Al-Taie A., Sancar M., Midi I. (2025). Impact of clinical pharmacist interventions on medication administration via enteral feeding tubes in a neurology ward: A pre- and post-educational prospective study. Front. Pharmacol..

[21] Dashti-Khavidaki S., Badri S., Eftekharzadeh S.Z., Keshtkar A., Khalili H. (2012). The role of clinical pharmacist to improve medication administration through enteral feeding tubes by nurses. Int. J. Clin. Pharm..

[22] European Medicines Agency Quality of Medicines Questions and Answers: Part 2. Administration of Oral Immediate Release Medicinal Products Through Enteral Feeding Tubes. https://www.ema.europa.eu/en/human-regulatory-overview/research-development/scientific-guidelines/quality-medicines-questions-answers-introduction/quality-medicines-questions-answers-part-2.

[23] Food and Drug Administration (2022). Oral Drug Products Administered via Enteral Feeding Tube: In Vitro Testing and Labeling Recommendations.

[24] Hu S., González N.N., Walsh J., Hermans E., Rassu G., Salunke S. (2025). Paediatric formulation challenges for enteral feeding tube administration—Current understanding and future directions. Adv. Drug Deliv. Rev..

[25] U.S Food and Drug Admnistration (2006). Quality Systems Approach to Pharmaceutical Current Good Manufacturing Practice Regulations.

[26] Yu L.X. (2008). Pharmaceutical Quality by Design: Product and Process Development, Understanding, and Control. Pharm. Res..

[27] Juran J.M. (1992). Juran on Quality by Design: The New Steps for Planning Quality into Goods and Services.

[28] International Council for Harmonisation of Technical Requirements for Pharmaceuticals for Human Use (ICH) (2023). ICH Harmonised Guideline Q9(R1): Quality Risk Management.

[29] International Council for Harmonisation of Technical Requirements for Pharmaceuticals for Human Use (ICH) (2009). ICH Guideline Q8 (R2): Pharmaceutical Development.

[30] International Council for Harmonisation of Technical Requirements for Pharmaceuticals for Human Use (ICH) (2008). ICH Harmonised Tripartite Guideline Q10: Pharmaceutical Quality System.

[31] International Conference on Harmonisation of Technical Requirements for Registration of Pharmaceuticals for Human Use (ICH) (2009). Harmonised Tripartite Guideline: Pharmaceutical Development Q8 (R2).

[32] Yang S., Hu X., Zhu J., Zheng B., Bi W., Wang X., Wu J., Mi Z., Wu Y. (2025). Aspects and Implementation of Pharmaceutical Quality by Design from Conceptual Frameworks to Industrial Applications. Pharmaceutics.

[33] Duarte J.G., Duarte M.G., Piedade A.P., Mascarenhas-Melo F. (2025). Rethinking Pharmaceutical Industry with Quality by Design: Application in Research, Development, Manufacturing, and Quality Assurance. AAPS J..

[34] Amidon G.L., DeBrincat G.A., Najib N. (1991). Effects of Gravity on Gastric Emptying, Intestinal Transit, and Drug Absorption. J. Clin. Pharmacol..

[35] Gibson M., Carmody A., Weaver R. (2018). Development and Manufacture of Drug Product. Pharmaceutical Quality by Design.

[36] Boullata J.I. (2021). Enteral Medication for the Tube-Fed Patient: Making This Route Safe and Effective. Nutr. Clin. Pract..

[37] White R., Bradnam V. (2015). Handbook of Drug Administration via Enteral Feeding Tubes.

[38] Pahsini K., Marinschek S., Khan Z., Urlesberger B., Scheer P.J., Dunitz-Scheer M. (2018). Tube dependency as a result of prematurity. J. Neonatal Perinat. Med..

[39] Roumeliotis N., Pullenayegum E., Rochon P., Taddio A., Parshuram C. (2020). A modified Delphi to define drug dosing errors in pediatric critical care. BMC Pediatr..

[40] Kim K.S., Cho H.J., Din F.U., Cho J.H., Choi H.G. (2025). Surfactant-Particle Engineering Hybrids: Emerging Strategies for Enhancing Solubility and Oral Bioavailability of Poorly Water-Soluble Drugs. Pharmaceutics.

[41] Brits M., Liebenberg W., de Villiers M.M. (2010). Characterization of polymorph transformations that decrease the stability of tablets containing the WHO essential drug mebendazole. J. Pharm. Sci..

[42] Karkossa F., Bading A., Klein S. (2024). What to consider for successful administration of oral liquids via enteral feeding tubes? a case study with paediatric ibuprofen suspensions. Int. J. Pharm..

[43] Blaszczyk A., Brandt N., Ashley J., Tuders N., Doles H., Stefanacci R.G. (2023). Crushed Tablet Administration for Patients with Dysphagia and Enteral Feeding: Challenges and Considerations. Drugs Aging.

[44] Casas-Augustench P., Salas-Salvadó J. (2009). Viscosity and flow-rate of three high-energy, high-fibre enteral nutrition formulas. Nutr. Hosp..

[45] Elia M., Crozier C., Martin S., Neale G. (1984). Flow and aspiration of artificial feeds through nasogastric tubes. Clin. Nutr..

[46] Walsh J., Hermans E., Salunke S. (2023). Navigating the complexities of medicating paediatric patients: Insights from an enteral feeding tube workshop. Eur. J. Pharm. Biopharm..

[47] Wilson S., Farabaugh J., Liu Y., Liu Z., Meyers R., Santangelo M., Thompson K. (2024). Oral Drug Product Administration via Enteral Feeding Tubes: In Vitro Testing. AAPS J..

[48] Blumenstein I., Shastri Y.M., Stein J. (2014). Gastroenteric tube feeding: Techniques, problems and solutions. World J. Gastroenterol..

[49] Pessoa Farias M.E., Lima Mesquita V., Dantas S.M.C., Silva A.R.A., Ayala A.P., de Oliveira Y.S., Beserra M.P.P., Fonteles M.M.d.F., Oliveira C.L.C.G. (2025). Maintaining the stability of furosemide tablets during enteral feeding tube delivery using PVC and non-PVC tubes, in accordance with hospital protocols. Eur. J. Hosp. Pharm..

[50] Beckwith M.C., Feddema S.S., Barton R.G., Graves C. (2004). A Guide to Drug Therapy in Patients with Enteral Feeding Tubes: Dosage Form Selection and Administration Methods. Hosp. Pharm..

[51] Alodat L., Shahin W., Breik L., Allahham A. (2026). Stability of Medications Administered via Enteral Feeding Tubes: A Systematic Review. Clin. Transl. Sci..

[52] Larsen J.A. (2023). Enteral Nutrition and Tube Feeding. Applied Veterinary Clinical Nutrition.

[53] Rajkumar L., Chandy S., Bright H., Jiji V., Basker J., Gunaraj K. (2023). Importance of Appropriate Medication Administration in Patients with Nasogastric Feeding Tube. J. Pharm. Res..

[54] Reddick C.A., Greaves J.R., Flaherty J.E., Callihan L.E., Larimer C.H., Allen S.A. (2023). Choosing wisely: Enteral feeding tube selection, placement, and considerations before and beyond the procedure room. Nutr. Clin. Pract..

[55] Bourgault A.M., Ipe L., Weaver J., Swartz S., O’dea P.J. (2007). Development of evidence-based guidelines and critical care nurses’ knowledge of enteral feeding. Crit. Care Nurse.

[56] Yu M., Chen J., Zheng S., Wang H., He X. (2020). Reduce medication errors in tube feeding administration by establishing administration standards and standardizing operation procedures. Drugs Ther. Perspect..

[57] Whelan K., Hill L., Preedy V.R., Judd P.A., Taylor M.A. (2006). Formula delivery in patients receiving enteral tube feeding on general hospital wards: The impact of nasogastric extubation and diarrhea. Nutrition.

[58] Bifari N., Bifari I.N., Alharbi Y.A. (2024). Unraveling medication errors in enteral tube administration: A cross-sectional study in geriatric patients receiving home health care. Saudi Pharm. J..

[59] Alsaeed D., Furniss D., Blandford A., Smith F., Orlu M. (2018). Carers’ experiences of home enteral feeding: A survey exploring medicines administration challenges and strategies. J. Clin. Pharm. Ther..

[60] Lu H., Rosenbaum S. (2014). Developmental pharmacokinetics in pediatric populations. J. Pediatr. Pharmacol. Ther..

[61] Patel S., Huang M., Miliara S. (2025). Understanding Treatment Adherence in Chronic Diseases: Challenges, Consequences, and Strategies for Improvement. J. Clin. Med..

[62] Bankhead R., Boullata J., Brantley S., Corkins M., Guenter P., Krenitsky J., Lyman B., Metheny N.A., Mueller C., Robbins S. (2009). Enteral nutrition practice recommendations. J. Parenter. Enter. Nutr..

[63] European Medicines Agency (EMA) (2021). Note for Guidance on In-Use Stability Testing of Human Medicinal Products (CPMP/QWP/2934/99).

[64] Mital A., Kilbom A. (1992). Design, selection and use of hand tools to alleviate trauma of the upper extremities: Part I—Guidelines for the practitioner. Int. J. Ind. Ergon..

[65] Klang M., Graham D., McLymont V. (2010). Warfarin bioavailability with feeding tubes and enteral formula. J. Parenter. Enter. Nutr..

[66] NPPG Executive Committee (2022). Administration of Medicines Using ENFit Syringes.

[67] van Welie S., Wijma L., Beerden T., van Doormaal J., Taxis K. (2016). Effect of warning symbols in combination with education on the frequency of erroneously crushing medication in nursing homes: An uncontrolled before and after study. BMJ Open.

[68] Galmarini E., Marciano L., Schulz P.J. (2024). The effectiveness of visual-based interventions on health literacy in health care: A systematic review and meta-analysis. BMC Health Serv. Res..

[69] Hodson L., Ovesen J., Couch J., Hirst D., Lawson C., Lentz T.J., MacKenzie B., Mead K., National Institute for Occupational Safety and Health (NIOSH) (2023). Managing Hazardous Drug Exposures: Information for Healthcare Settings.

[70] U.S. Pharmacopeial Convention General Chapter <800>: Hazardous Drugs—Handling in Healthcare Settings. https://www.usp.org/compounding/general-chapter-hazardous-drugs-handling-healthcare.

[71] Sommerfeldt J., Sartorius H., von Sarnowski B., Klein S., Ritter C.A. (2024). Drug administration via feeding tubes-a procedure that carries risks: Systematic identification of critical factors based on commonly administered drugs in a cohort of stroke patients. Eur. J. Clin. Pharmacol..

[72] Stefanski B.S., Heuberger R.A. (2025). A Scoping Review of Interventions to Reduce Feeding Tube-Related Medication Errors. Gastroenterol. Nurs..

[73] Pereira R.A., Bonacim C.A.G., da Costa L.R.M., Rigobello M.C.G., de Souza F.B., Grande M.M., Gimenes F.R.E. (2023). Impact of a quality improvement programme on the preparation and administration of medications via a nasoenteral feeding tube: 2014-2019 intervention study. BMJ Open Qual..

[74] Külekci E., Iyigün E. (2025). Effectiveness of a checklist for enteral medication administration: A randomized controlled trial. Nurs. Crit. Care.

[75] van den Bemt P.M., Cusell M.B., Overbeeke P.W., Trommelen M., van Dooren D., Ophorst W.R., Egberts A.C. (2006). Quality improvement of oral medication administration in patients with enteral feeding tubes. Qual. Saf. Health Care.

[76] Cavagna P., Bizet S., Fieux F., Houillez E., Chirk C., Zulian C., Perreux J., Fernandez C., Lescot T., Antignac M. (2022). Assessment of Good Practice Guidelines for Administration of Drugs via Feeding Tubes by a Clinical Pharmacist in the Intensive Care Unit. Crit. Care Nurse.

[77] Santangelo M., Wood J.A., Barnett K.L., Wooding F.G.G., Bartlett J.A. (2022). In Vitro Assessment for Dose Preparation and Simulated Administration of Azithromycin Suspensions via Enteral Feeding Tubes. Hosp. Pharm..

[78] Samuels S., Denisenko D., Abdel-Rahman S., Fletcher E.P. (2026). Drug Administration via Enteral Feeding Tubes: A Landscape Analysis of Information in Drug Product Labeling. J. Clin. Pharmacol..

[79] Garrison C.M. (2018). Enteral Feeding Tube Clogging: What Are the Causes and What Are the Answers? A Bench Top Analysis. Nutr. Clin. Pract..

[80] FDA. Food and Drug Administration (2012). CDER Patient-Focused Drug Development Webpage. https://www.fda.gov/drugs/development-approval-process-drugs/cder-patient-focused-drug-development.

[81] Food and Drug Administration (FDA) (2020). Patient-Focused Drug Development: Collecting Comprehensive and Representative Input Guidance for Industry, Food and Drug Administration Staff, and Other Stakeholders.

[82] International Council for Harmonisation of Technical Requirements for Pharmaceuticals for Human Use (ICH) (2020). ICH Reflection Paper on Proposed ICH Guideline Work to Advance Patient Focused Drug Development.

