# Supplementary material for: Introducing the Quality Target Administration (QTAP) Profile for Enteral Feeding Tube Medications
Source: Pharmaceutics. 2026 Aug 29;18(9):1089. doi: 10.3390/pharmaceutics18091089 (PMC13610307; doi:10.3390/pharmaceutics18091089)
Supplement: Supplementary file 1 [file pharmaceutics-18-01089-s001.zip › pharmaceutics-4468035-supplementary.pdf]

Introducing the Quality Target Administration (QTAP) Profile for Enteral Feeding Tube Medications

Supplementary Material

Table S1: Comprehensive Administration Attributes for Three EFT Scenarios

*Complete attribute lists generated through Phase 2 systematic identification across five QTAP categories. Attributes marked with asterisks (*) were subsequently designated as CAAs following Phase 3 criticality assessment. *

SCENARIO 1: READY-TO-USE ORAL SUSPENSION (Total: 26 attributes)

Category	#	Attribute	Description/Specification
**Dose Delivery Performance**	1*	Suspension uniformity after shaking	Homogeneity of drug distribution throughout bottle after specified shaking protocol
	2*	Dose measurement accuracy	Accuracy of dose measurement using provided/recommended oral syringe; ability to measure fractional volumes for weight-based dosing
	3*	Dose recovery from tube	Percentage of measured dose successfully delivered to patient accounting for tube adherence and flush effectiveness
	4	Dose precision across multiple administrations	Reproducibility of delivered dose when same caregiver administers multiple times
	5	Dose precision across different users	Consistency of delivered dose when different caregivers (varied training/experience) administer
	6	Suspension uniformity persistence during administration	Duration that suspension remains adequately uniform after shaking (administration window)
	7	Dose consistency first-to-last dose	Maintenance of dose accuracy throughout multi-dose bottle use period (first dose vs. 50th dose)
	8	Redispersibility after storage	Ease and completeness of resuspending settled product after hours/days between doses
**Flow & Tube Compatibility**	9*	Tube blockage risk - 8Fr tubes	Probability of tube occlusion when administered through smallest pediatric tubes (8 French)
	10	Tube blockage risk - 10-12Fr tubes	Blockage probability in mid-range pediatric tubes
	11	Tube blockage risk - 14Fr tubes	Blockage probability in larger pediatric tubes
	12*	Flow time through 8Fr tubes	Duration required for dose to transit tube at typical administration rates
	13	Flow time through 12Fr tubes	Transit time through mid-range tubes

Category	#	Attribute	Description/Specification
	14	Flow time through 14Fr tubes	Transit time through larger tubes
	15	Plunger force requirements	Force required to depress syringe plunger during administration; must be achievable by caregivers with varied strength
	16	Product adherence to tube materials - polyurethane	Percentage of dose retained on polyurethane tube walls after administration and flushing
	17	Product adherence to tube materials - silicone	Percentage retained on silicone tubes
	18	Compatibility with enteral formula residue	Absence of precipitation, gel formation, or phase separation when contacting residual enteral feeds
	19	Viscosity appropriateness	Balance between viscosity high enough for suspension stability and low enough for tube flow
**Preparation & Process**	20	Shaking requirements - inversions needed	Number and vigor of bottle inversions required to achieve adequate resuspension
	21	Shaking requirements - duration	Time duration of shaking required
	22*	Flush volume requirements	Volume of flush water needed to achieve ≥95% dose recovery; must balance completeness vs. fluid restrictions
	23	Pre-flush requirements	Whether tube must be flushed before administration to clear residual feeds
	24	Total administration time	Complete time from accessing bottle through flush completion
	25	Equipment requirements	Specific oral syringe types/sizes, flush water (sterile vs. tap), accessibility
**User Interface & Error Prevention**	26*	Instructions for Use (IFU) clarity	Comprehensibility of shaking, measuring, and flushing instructions for varied health literacy levels
	27	Visual confirmation of adequate resuspension	Ability of caregivers to visually assess whether shaking achieved uniform suspension
	28	Oral syringe graduation readability	Clarity of volume markings; ability to read fractional volumes (e.g., 3.7 mL)
	29	Bottle opening accessibility	Ease of inserting oral syringe into bottle opening for dose withdrawal
	30	Tactile feedback during administration	Plunger resistance providing feedback about flow/blockage
**Safety & Product Integrity**	31	Microbiological stability - multi-dose period	Preservative system effectiveness over 30-day in-use period
	32	Contamination prevention during withdrawal	Minimization of contamination risk when inserting syringe into bottle repeatedly

Category	#	Attribute	Description/Specification
	33	Child-resistant closure functionality	Cap prevents child access while remaining openable by caregivers
	34	In-use chemical stability	Maintenance of drug potency during repeated bottle opening/closing over use period
	35	Light exposure protection	Amber bottle protection of photosensitive actives during storage and use

---

SCENARIO 2: CRUSHED TABLET (OFF-LABEL) (Total: 32 attributes)

Category	#	Attribute	Description/Specification
**Dose Delivery Performance**	1*	Crushing completeness	Extent to which tablet is reduced to fine particles without large fragments that could block tubes
	2	Particle size after crushing	Distribution of particle sizes achieved through crushing; varies with crushing method and equipment
	3*	Dose recovery from crushing device	Percentage of dose successfully transferred from mortar/pestle or crusher to mixing container
	4*	Dose recovery from mixing container	Percentage transferred from mixing container to administration syringe
	5*	Cumulative dose recovery	Overall percentage delivered to patient accounting for all transfer steps (typically 70-85% for crushed tablets)
	6	Particle suspension stability in water	Duration crushed particles remain suspended in water slurry before settling
	7	Particle resuspendability in slurry	Ability to resuspend settled particles if administration is delayed
	8	Dose precision across caregivers	Consistency of delivered dose given high technique variability in crushing and transfers
	9	Dose measurement accuracy in slurry	Accuracy of measuring slurry volume containing suspended particles
**Flow & Tube Compatibility**	10*	Tube blockage risk - crushed particles	Probability of blockage from incompletely crushed fragments or aggregated particles; substantially higher than formulated suspensions
	11	Slurry viscosity variability	Inconsistency in slurry viscosity depending on water volume added and mixing thoroughness
	12	Flow time variability	Variation in flow time due to inconsistent particle size and slurry preparation
	13	Particle settling during administration	Sedimentation of particles during the 2-5 minute administration, potentially leaving dose in syringe
	14	Compatibility with residual enteral feeds	Risk of precipitation or aggregation when crushed tablet slurry contacts enteral formula residue

Category	#	Attribute	Description/Specification
	15	Tube material adherence	Product loss to tube walls; may be higher for crushed tablets than formulated products
**Preparation & Process**	16*	Crushing method standardization	Variability in crushing technique (mortar/pestle, tablet crusher, improvised methods) affecting outcomes
	17	Crushing equipment variability	Differences in equipment available across settings (hospital pharmacy vs. home)
	18	Crushing adequacy assessment	Absence of clear endpoint - caregivers cannot easily determine when crushing is "adequate"
	19	Transfer step count	Multiple transfer steps (crush → mix container → syringe) each introducing loss
	20	Mixing technique requirements	Thoroughness of mixing crushed powder with water to create administrable slurry
	21	Water volume for mixing	Amount of water needed to create slurry; too little = thick paste, too much = large flush needed
	22*	Total preparation time	8-10 minutes typical for crushing, transferring, mixing, administering, flushing; high caregiver burden
	23	Equipment cleaning between medications	Need to clean crushing/mixing equipment when administering multiple medications sequentially
	24*	Flush volume requirements	Higher flush volumes needed due to greater tube adherence and particle settling; conflicts with fluid restrictions
**User Interface & Error Prevention**	25*	Crushing instruction clarity	Guidance on crushing method, equipment, adequacy assessment; often absent in labeling
	26*	Multi-step error potential	Cumulative error risk across crushing, multiple transfers, mixing, administration steps
	27	Visual assessment limitations	Difficulty visually confirming adequate crushing or complete transfer
	28	Technique-dependent outcomes	Heavy reliance on caregiver skill and experience for quality outcomes
	29	Training requirements	Need for hands-on training in crushing technique; rarely provided systematically
**Safety & Product Integrity**	30*	Powder exposure during crushing	Caregiver inhalation or dermal exposure to drug powder; critical for hazardous drugs (chemotherapy, teratogens)
	31	Cross-contamination of equipment	Residual drug in crushing devices contaminating subsequent medications
	32*	Appropriateness of crushing - formulation suitability	Whether tablet formulation is suitable for crushing (immediate-release OK; modified-release, enteric-coated, hazardous may be contraindicated)
	33	Loss of modified-release characteristics	Crushing destroys controlled-release mechanisms, potentially causing dose dumping

Category	#	Attribute	Description/Specification
	34	Loss of enteric coating protection	Crushing exposes acid-labile drugs to gastric acid or removes GI protection
	35	Moisture exposure during preparation	Drug degradation from moisture exposure during mixing with water
	36	Air/light exposure	Degradation of sensitive actives during extended preparation process

---

SCENARIO 3: ORAL SOLUTION (Total: 20 attributes)

Category	#	Attribute	Description/Specification
**Dose Delivery Performance**	1*	Dose measurement accuracy	Critical for concentrated solutions (50 mg/mL); small volume errors = large dose errors
	2	Measurement precision requirements	Need for precision measuring devices (0.1 mL graduations) for small pediatric doses
	3*	Dose completeness	Minimizing residual volume in syringe and tube dead space
	4	Dose consistency throughout bottle	Solution uniformity maintained throughout multi-dose use
	5	Evaporation effects	Potential concentration changes if bottle left open or stored improperly
**Flow & Tube Compatibility**	6	Rapid flow characteristics	Very low viscosity ensures rapid transit through tubes
	7	Flow time through 6Fr tubes	Transit time through smallest neonatal tubes
	8	Flow time through 8-12Fr tubes	Transit time through standard pediatric tubes
	9*	Osmolality	Hyperosmolar solutions (>450 mOsm/kg) risk feeding intolerance, NEC in neonates; must be isotonic or near-isotonic
	10	pH for GI tolerance	pH compatibility with neonatal GI tract; very acidic or alkaline solutions poorly tolerated
	11*	Tube material compatibility	Compatibility with polyurethane, silicone, PVC; minimal drug adsorption
	12	Compatibility with enteral formulas	No precipitation when mixed with residual feeds
**Preparation & Process**	13	Minimal preparation steps	Measure and administer; no shaking, crushing, or mixing required
	14*	Flush volume requirements	Critical in fluid-restricted neonates; balance dose recovery vs. fluid intake limits
	15	Pre-flush requirements	Whether tube requires flushing before dose to clear feeds

Category	#	Attribute	Description/Specification
	16	Total administration time	Typically 1-2 minutes; minimal burden
	17	Equipment requirements	Precision oral syringe (1 mL or smaller for neonates) with clear graduations
**User Interface & Error Prevention**	18	Measurement precision for small volumes	Ability to accurately measure 0.3-0.8 mL doses common in neonatal weight-based dosing
	19*	Concentration error prevention	Risk of decimal point errors, dilution errors, or confusion between mg and mL
	20	Syringe selection guidance	Instructions specifying appropriate syringe size for dose range
	21	Visual clarity of solution	Clear solution enables detection of particulates or precipitation
	22	Graduated device readability	Clear markings on small-volume syringes
**Safety & Product Integrity**	23*	Accidental concentration errors	Risk of administering undiluted when dilution intended, or vice versa
	24	Hyperosmolar complication risk	GI intolerance, osmotic diarrhea, NEC risk if osmolality too high
	25	In-use chemical stability	Maintenance of potency during multi-dose use period
	26	Microbiological stability	Preservative effectiveness for multi-dose solutions
	27	Child-resistant packaging	Protection against accidental pediatric ingestion
	28	Container tip design	Easy syringe insertion for dose withdrawal without spillage

---

Notes:

1. **Asterisks (*)** denote attributes that qualified as CAAs following Phase 3 criticality assessment detailed in manuscript Table 3.
2. **Attribute counts:**
   - Scenario 1 (Suspension): 35 total attributes, 6 CAAs
   - Scenario 2 (Crushed Tablet): 36 total attributes, 7 CAAs
   - Scenario 3 (Solution): 28 total attributes, 5 CAAs
3. **Domain distribution** varies by scenario complexity:
   - Crushed tablet has most attributes in Preparation & Process (9 items) and Safety (7 items) reflecting manipulation complexity
   - Solution has fewest overall attributes due to minimal preparation requirements
   - Suspension has balanced distribution reflecting formulation-dependent challenges (uniformity, flow) combined with technique dependencies (shaking, flushing)
4. **Technique-dependent attributes** (where quality depends on user execution rather than product characteristics) are most numerous in Scenario 2 (crushed tablet), explaining the "HIGH" overall risk rating in Table 6.

5. These attributes represent systematic Phase 2 output prior to criticality filtering. The focused CAA lists in manuscript Table 9 resulted from Phase 3 risk-based prioritization of these comprehensive attributes.

Table S1 presents the complete administration attributes generated through QTAP Phase 2 systematic identification for three representative EFT scenarios. Attributes span five categories: dose delivery performance, flow and tube compatibility, preparation and process requirements, user interface and error prevention, and safety and product integrity. The attributes demonstrate that attribute quantity and composition are dosage form-dependent, with off-label manipulation (crushed tablet) generating substantially more attributes, particularly technique-dependent attributes, than purpose-designed formulations. Attributes subsequently designated as CAAs through Phase 3 criticality assessment are marked with asterisks and detailed in manuscript Table 6
